# The venomous cocktail of the vampire snail *Colubraria reticulata* (Mollusca, Gastropoda)

**DOI:** 10.1186/s12864-015-1648-4

**Published:** 2015-06-09

**Authors:** Maria Vittoria Modica, Fabrizio Lombardo, Paolo Franchini, Marco Oliverio

**Affiliations:** Department of Biology and Biotechnologies “C. Darwin”, Sapienza University, I-00185 Rome, Italy; Department of Public Health and Infectious Diseases, Sapienza University, I-00185 Rome, Italy; Department of Biology, University of Konstanz, D-78745 Konstanz, Germany

**Keywords:** *Colubraria*, Neogastropoda, Transcriptome, NGS, Hematophagy, Kunitz/TFPI peptides, vWFA, Salivary glands, Mid-oesophagus

## Abstract

**Background:**

Hematophagy arose independently multiple times during metazoan evolution, with several lineages of vampire animals particularly diversified in invertebrates. However, the biochemistry of hematophagy has been studied in a few species of direct medical interest and is still underdeveloped in most invertebrates, as in general is the study of venom toxins. In cone snails, leeches, arthropods and snakes, the strong target specificity of venom toxins uniquely aligns them to industrial and academic pursuits (pharmacological applications, pest control etc.) and provides a biochemical tool for studying biological activities including cell signalling and immunological response. Neogastropod snails (cones, oyster drills etc.) are carnivorous and include active predators, scavengers, grazers on sessile invertebrates and hematophagous parasites; most of them use venoms to efficiently feed. It has been hypothesized that trophic innovations were the main drivers of rapid radiation of Neogastropoda in the late Cretaceous.

We present here the first molecular characterization of the alimentary secretion of a non-conoidean neogastropod, *Colubraria reticulata*. Colubrariids successfully feed on the blood of fishes, throughout the secretion into the host of a complex mixture of anaesthetics and anticoagulants. We used a NGS RNA-Seq approach, integrated with differential expression analyses and custom searches for putative secreted feeding-related proteins, to describe in detail the salivary and mid-oesophageal transcriptomes of this Mediterranean vampire snail, with functional and evolutionary insights on major families of bioactive molecules.

**Results:**

A remarkably low level of overlap was observed between the gene expression in the two target tissues, which also contained a high percentage of putatively secreted proteins when compared to the whole body. At least 12 families of feeding-related proteins were identified, including: 1) anaesthetics, such as ShK Toxin-containing proteins and turripeptides (ion-channel blockers), Cysteine-rich secretory proteins (CRISPs), Adenosine Deaminase (ADA); 2) inhibitors of primary haemostasis, such as novel vWFA domain-containing proteins, the Ectonucleotide pyrophosphatase/phosphodiesterase family member 5 (ENPP5) and the wasp Antigen-5; 3) anticoagulants, such as TFPI-like multiple Kunitz-type protease inhibitors, Peptidases S1 (PS1), CAP/ShKT domain-containing proteins, Astacin metalloproteases and Astacin/ShKT domain-containing proteins; 4) additional proteins, such the Angiotensin-Converting Enzyme (ACE: vasopressive) and the cytolytic Porins.

**Conclusions:**

*Colubraria* feeding physiology seems to involve inhibitors of both primary and secondary haemostasis, anaesthetics, a vasoconstrictive enzyme to reduce feeding time and tissue-degrading proteins such as Porins and Astacins. The complexity of *Colubraria* venomous cocktail and the divergence from the arsenal of the few neogastropods studied to date (mostly conoideans) suggest that biochemical diversification of neogastropods might be largely underestimated and worth of extensive investigation.

**Electronic supplementary material:**

The online version of this article (doi:10.1186/s12864-015-1648-4) contains supplementary material, which is available to authorized users.

## Background

Hematophagy arose independently multiple times during metazoan evolution, leading to the appearance of several lineages of vampire animals that exploit hosts’ blood, a renewable and nutrient-rich source. Beside vertebrates (bats and lampreys), in invertebrates hematophagous parasites are found amongst platyhelminths (flukes), nematodes (hookworms), annelids (leeches), molluscs and in arthropods with more than 14,000 hematophagous species, ranging from acarines (ticks) to insects (mosquitoes, sandflies, bugs, fleas, horseflies, midges, moths) and crustaceans (sea lice, fish lice) [1, 2].

Adaptations to blood-feeding life style include behavioral traits, fundamental for host location, anatomical features, often including specialized mouthparts and biochemical specialization, with the production of complex secretions (generally including molecules acting as anti-hemostatic, anesthetic and anti-inflammatory) in specialized glands [2, 3].

The biochemical bases of blood feeding have been investigated to date only in a reduced number of medically important species, either for their therapeutic use, as is the case of leeches [4, 5], or for their role as vectors of deadly diseases such as ticks and insects [6–8] that are undoubtedly the most explored group. These investigations revealed that hematophagous animals have convergently recruited a series of molecules acting on different physiological stages of host hemostasis, including vasoconstriction, formation of both platelet plugs and fibrin clots and fibrinolysis [3, 5, 7, 9]. Platelet aggregation is the first stage of hemostasis, and is activated by collagen, thrombin, ADP and thromboxane A [9]. Secondary hemostasis, or blood coagulation cascade, consists of a series of enzymatic reaction where coagulation factors (inactive proenzymes) are converted in their active forms, generally proteases, that in turn activate the next proenzyme in the cascade, converging on a final common pathway concluded by thrombin that converts fibrinogen in fibrin clot. Generally speaking, hematophagous animals target the hemostatic system of the host at the level of both primary and secondary hemostasis, producing a number of proteins of different molecular masses, that act interacting with several targets and exert a variety of inhibitory mechanisms [3, 5, 7, 9].

**Fig. 1:**
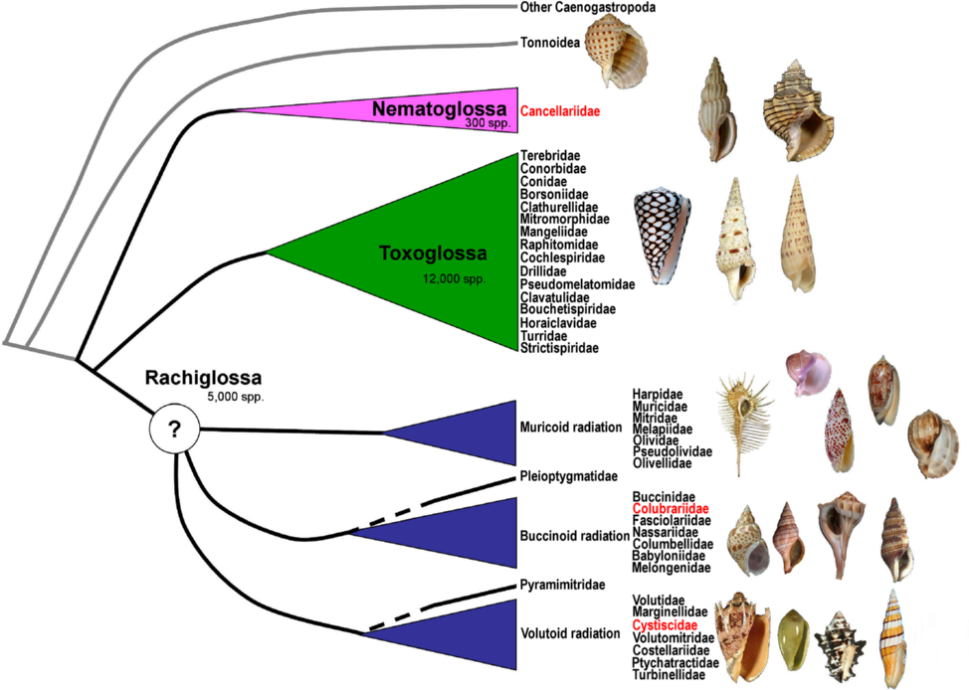
Phylogenetic relationships among the major lineages of Neogastropoda. The phylogenetic tree was built according to molecular- and morphology-based analyses [14-16, 24, 127, 128]: solid lines define branches that are supported by molecular data while dashed lines define branches supported by morphological data. Families comprising hematophagous species (Cancellariidae, Colubrariidae and Cystiscidae) are in red. Shells of representative species for each major lineage are pictured (not to scale)

Among the less studied hematophagous groups, vampire snails belong to three lineages of Neogastropoda, namely Cancellariidae [10], Colubrariidae [11], and Cystiscidae [12, 13] that according to the available phylogenetic framework for neogastropods [14-16] evolved hematophagy independently (Fig. 1). Neogastropoda include many familiar molluscs, such as cone snails (Conidae), purple dye snails (Muricidae), mud snails (Nassariidae), olive snails (Olividae), oyster drills (Muricidae), tulip shells (Fasciolariidae), and whelks (Buccinidae). The vast majority of neogastropods are carnivorous, with a degree of predatory activity that varies from actively seeking prey, scavenging, grazing on sessile invertebrates, or hematophagous parasitism. According to the fossil record, the adaptive radiation of neogastropods has been particularly rapid [17] and was accompanied by the diversification of their predatory lifestyles including a number of different trophic strategies. It has been thus hypothesized [17, 18] that evolutionary innovations related to the biochemistry of feeding were the main drivers of the rapid neogastropod radiation in the late Cretaceous, but such innovations have been investigated to date only in a few species of the venomous Conoidea [19-22].

**Fig. 2:**
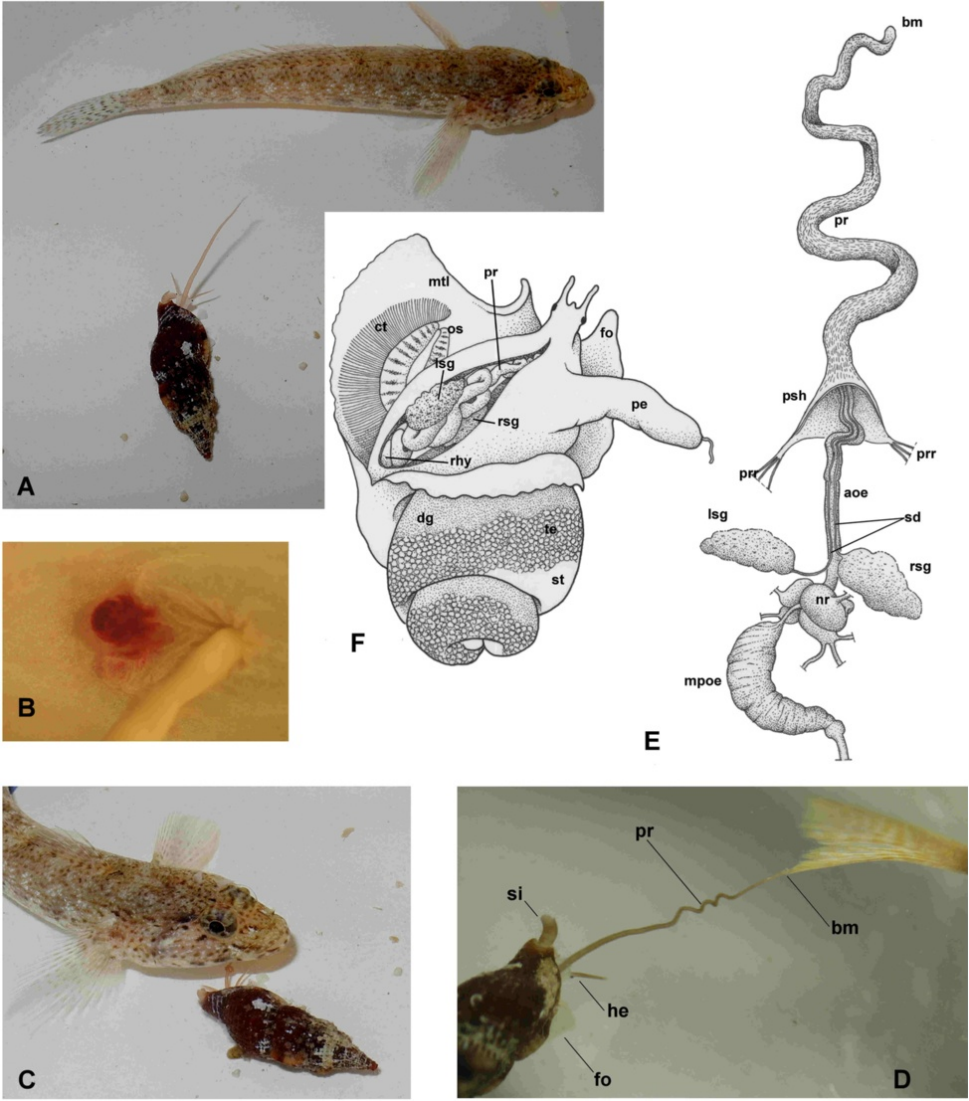
Anatomy and feeding activity of *Colubraria reticulata*. **a**: a specimen of *C. reticulata* crawling towards a *Gobius bucchichi* (Perciformes: Gobiidae), with the proboscis extended. The fish appear to be semi-anesthetized. **b**: circular bleeding wound found after *C. reticulata* detachment behind the pelvic fin of *Uranoscopus scaber* (Perciformes: Uranoscopidae). **c**: *C. reticulata* probing the sub-opercular area of *G. bucchichi*; **d**: *C. reticulata* feeding on the caudal fin of *G. bucchichi*; **e**: Dissected foregut of *C. reticulata*, from the proboscis tip to the mid-posterior oesophagous **f**: Body of C. *reticulata*. Mantle and body wall are medially dissected to show mantle organs and organization of the foregut. Abbreviations: an, anus; aoe, anterior oesophagus; bm, buccal mass; ct, ctenidium; dg, digestive gland; fo, foot; go, gonad; he, head; hyg, hypobranchial gland; lsg, left salivary gland; mo, mouth; mpoe, mid-posterior oesophagus; mtl, mantle; nf, nerve fibre; nr, nerve ring; os, osphradium; pa, proboscideal artery; p, penis; pr, proboscis; prr, proboscis retractor muscle; psh, proboscis sheath; re, rectum; rsg, right salivary gland; ry, rhynchodaeum; sd, salivary gland duct; si, siphon; st, stomach

The buccinoidean family Colubrariidae includes two dozen marine shallow-water species, probably all hematophagous, that inhabit rocky and coral environments in tropical, subtropical and temperate seas. At least six species (25 % of the family) have been documented in a parasitic association with different species of fish, mainly belonging to the family Scaridae [11, 23]. Both females and males are hematophagous: the blood meal is not related to any particular physiological state, and may occur at any time, although in nature is generally observed during the night time [11, 23]. The association is not species-specific, but seem to require a resting or slow moving fish (Modica & Oliverio, unpublished observation). Colubrariidae use a long and thin proboscis to feed on the blood of fishes [11, 23] (Modica & Oliverio, unpublished observation; Fig. 2). Specimens of the genus *Colubraria* Schumacher, 1817, which accounts for most of the species diversity of colubrariids, can extend their proboscis to a length exceeding three times the shell length. Initially, the extended proboscis contacts the skin of the prey, then, it gains access to the blood vessels of the fish. The radula is extremely minute in *Colubraria*, so that the genus was considered radula-less until very recently [15]. While a scraping action by such a reduced radula is still possible, the wounds observed in fishes seem to indicate the involvement of bioactive secretions (Fig. 2b). The snail then apparently takes advantage of the blood pressure of the fish to ingest its meal, congruently with the thin structure of the proboscis that is unlikely to exert an active suction [15]. Experimental observations on different *Colubraria* species (Modica and Oliverio, unpublished) suggest that adaptation to hematophagy involves the use of anesthetic and anticoagulant compounds. In fact, open wounds on the fish skin, often accompanied by bleeding, are evident on the fish skin after *Colubraria* detaching (Fig. 2b). Moreover, the fishes being fed on are generally resting and do not seem to be disturbed by the hematophagous activity of the colubrariid snail. It should be noted that anesthetization is reversible, as the fish usually recovers its full mobility in a few minutes after the snail disengages. This observation, indicating that the anaesthetic compounds used by the snail are not lethal to the prey, is in agreement with field observations that tropical colubrariids usually feed on fish sleeping in crevices of the reef (M. Oliverio unpublished observation, [11, 23]). *Colubraria* is a buccinoidean neogastropod, with some remarkable anatomical peculiarities, mainly regarding the structure of the anterior and mid-posterior oesophagous, described elsewhere for the type species *C. muricata* [15]. The anatomical structures responsible for anesthetic secretion have not been yet identified, however given the Colubrariidae foregut anatomy (Fig. 2e-f) anticoagulants are likely produced in the salivary glands. While the absence of accessory salivary glands, gland and valve of Leiblein are features quite common amongst buccinoideans [24-26], the glandularization of the posterior portion of the mid-oesophagous is a peculiar derived characteristic of colubrariids [15], worth of investigation since it may be related to the hematophagous lifestyle and it potentially shares some homology degrees with the toxoglossan venom glands [24-26].

We present here the first molecular characterization of the alimentary secretions of a non-conoidean neogastropod, describing the salivary glands and mid-oesophagous transcriptomes of the hematophagous Mediterranean colubrariid *Colubraria reticulata*, with functional and evolutionary insights on major classes of bioactive molecules.

## Results and discussion

### General assembly characteristics

After sequencing and filtering we obtained a final dataset of 220,305,266 filtered reads that were used to build the transcriptome assembly (Additional file 1: Table S1).

Assembly produced a total of 144,380 contigs longer than 60 bp, ranging from 60 bp to 16,266 bp (median 230 bp); 56 % of the total contigs were longer than 200bp. A general annotation of the transcriptome using BLASTX [27] revealed 64,387 contigs with a significant similarity to sequences in the NR reference database (44.6 %), while 108,535 contigs (75 %) matched with functional protein domains in InterProScan [28] with at least one of the following search methods: SignalP, TMHMM, Pfam, SMART, Tigr, ProfileScan (Additional file 1: Table S2 and S3).

The total number of transcripts retrieved for *C. reticulata* transcriptome is congruent with previously reported datasets from other non-model species of invertebrates, including gastropods [29-31].

A total of 1437 transcripts possibly derive from transposable elements, possessing RT PFAM domains indicative of reverse transcriptase. The presence of these transcripts may indicate transposition activity taking place in the genome of *C. reticulata.*

Protein families (PFAM) that are enriched in the whole-body with respect to both target tissues (Additional file 1: Table S4; Additional file 2: Figure S1) include large multifunctional families such as Thrombospondin-, Kelch-, ARM-, and WD40-containing proteins, putative oxygen carriers containing globin and haemocyanin domains, protein with kinase and tyrosinase domains, HMG-box-containing proteins involved in DNA-regulation processes, immunity components such as complement and immunoglobulins, and sugar transporters. The iron-storage Ferritin proteins that in mollusks have been related with immunity, development and shell formation processes [32], and LEA (Late Embryogenesis Abundant) proteins that are thought to be involved in the response to drying and osmotic stresses in plants and nematodes [33] were also enriched in the whole body. Enrichment in the whole body involves also proteins containing a ShK toxin domain, whose expression is enhanced in the salivary subset as well (see below).

### Gene-expression profiling of glandular tissues

**Fig. 3:**
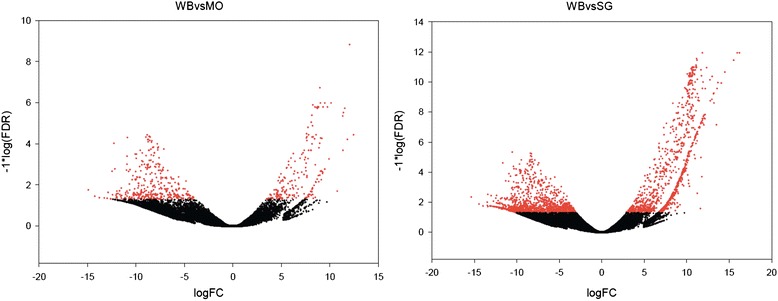
Tissue-specific gene expression. Volcano plots displaying the relative expression levels of transcripts in the mid-oesophagous (left) and salivary glands (right) versus the whole body. The x-axis represent the log2 of the expression ratio for each transcript of *Colubraria* transcriptome (tissue specific logCPM: whole body logCPM, where CPM stands for Counts Per Million reads); the y-axis represents the log10 of the p-value corrected for the false discovery rate. Red points represent transcripts with logFDR < 0.05 a greater than 3-folds difference in logCPM). Positive logFC values indicate transcripts enhanced in the tissue-specific subset, while negative values indicate transcripts enhanced in the whole body

Our NGS-based transcriptome analysis revealed that, with a conservative approach (logFC > =3, FDR < 0.05), 935 contigs showed enhanced expression in the salivary glands when compared to whole body while 184 contigs showed enriched expression in the mid-esophagus (Fig. 3; Additional file 1: Table S5, Table S6).

Among these, 378 and 93 contigs respectively did not display a significant similarity with known proteins in the NR database by BLAST search, corresponding to 40.3 % for salivary glands and 50.5 % for mid-oesophagus. This high rate of transcripts that were unidentifiable with a BLAST search is congruent with corresponding rates observed in the transcriptomic analyses of venom-producing glands of other taxa such as arthropods [34-36], where more than 30 % to more than 50 % of transcript resulted unknown. However, when we examined singularly the most overexpressed contigs (SI > 65 %, where SI is the normalized tissue-specific logFC value for each contig) with no BLAST, we were able to recover similarities for about half of them.

**Fig. 4:**
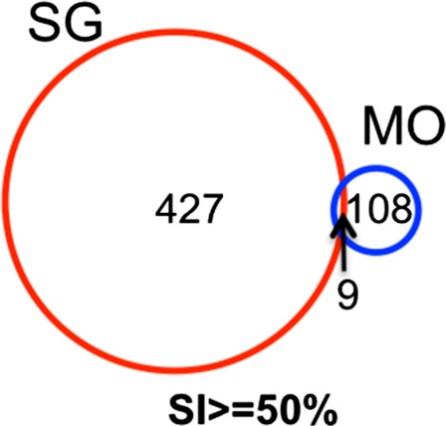
Enriched transcripts pairwise tissue comparison. Proportional Venn diagrams showing the comparison between salivary-specific and mid-oesophagous-specific transcripts, with a tissue-specificity index greater than 50 %. Overlap represents the number of transcripts whose expression is significantly enhanced in both tissues

The expression of only 32 contigs, 13 of which had no BLAST hits, was enhanced in both tissues. Limiting the analysis to contigs with a SI > =50 %, the overlap between the two tissue-specific subsets decreases to 9 contigs, two of which have no BLAST hit (Fig. 4; Additional file 1: Table S7).

**Fig. 5:**
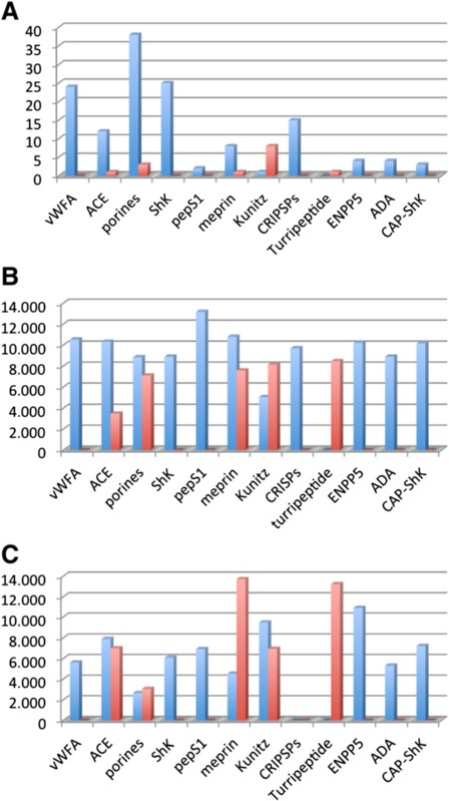
Comparison of tissue-specific expression of targeted protein classes. **a** Abundance of transcripts belonging to targeted venom protein classes in each tissue; **b** Median logFC of transcripts belonging to targeted venom protein classes in each tissue; **c** Median logCPM of transcripts belonging to targeted venom protein classes in each tissue. Mid-oesophageal transcripts in red, salivary transcripts in red

The most represented contigs of the tissue-specific subsets encode for proteins/domains that belong to different PFAM families (Fig. 5). Specifically, Kunitz-BPTI, FMRFamide-related peptides (FARPs), WD40 proteins and whey acidic proteins (WAP) are the protein domains that dominate the oesophageal subset, which is generally scarcely diversified, with only 7 families represented by more than 2 contigs (Additional file 1: Table S8). Salivary subset includes 50 families represented by more than 2 contigs, and is composed mainly by transcripts containing the following domains: ShK Toxin, von Willebrand domain A (vWA), metalloproteases (astacin and peptidase M14), prolyl-oligopeptidases (Peptidase S9) and RNA-binding proteins (KH). Our results indicate a higher complexity of the salivary gene expression profile: indeed, the most represented PFAM families include several potential feeding-related proteins (Additional file 1: Table S9). In both tissues a number of transcripts probably derive from transposable elements (24 in the salivary subset and 3 in the mid-oesophageal subset).

We refined the search of predicted signal peptides in both salivary- and oesophageal-enriched catalogues with the aim to better define the secretory nature of predicted proteins in these repertoires. The salivary (935 contigs) and the oesophageal (184 contigs) subsets were therefore manually translated (to retrieve only peptides starting with Met) and the probability to carry signal peptides was evaluated using two on-line prediction servers, SignalP and PrediSi [37, 38] (Table 1)

**Table 1:** Predicted number of secreted contigs. Number of secreted peptides predicted by SignalP and PrediSi servers for tissue-specific and whole-body enhanced contigs subsets. Percentages of secreted peptides over the total number of tissue-specific and whole-body enhanced contigs are also reported

	MO	SG	WB
Peptides	178	929	1048
SP	80	305	235
% SP/total	44.9	32.8	22.4

.

Putative proteins carrying a predicted signal sequence were found 80 (43.5 % of the total) and 305 (32.6 % of the total) analysing the oesophageal- and salivary-enriched catalogues, respectively. As expected, when the same procedure was applied to search for predicted signal peptides in a subset of contigs enriched in the whole body (a catalogue containing 1,048 contigs), a lower rate (22.4 %) of putative proteins carrying a signal peptide was predicted, pointing out the secretory profile of salivary and oesophageal repertoires.

The rate of predicted signal peptides in the *C. reticulata* secretory organs is consistent with previous observations in other hematophagous arthropods such as, for instance, the malaria mosquito *Anopheles gambiae,* the yellow fever mosquito *Aedes aegypti,* and the tick *Ixodes scapularis*, where respectively the 18 %, 22.1 % and 29 % of the salivary transcripts were predicted as secreted [39–41].

Custom search for putative feeding-related proteins in the tissue-specific subsets allowed the selection of members of the following protein families: a) vWFA-containing protein; b) porins; c) angiotensin-converting enzyme (ACE); d) TFPI-like Kunitz protease inhibitors; e) ectonucleotide pyrophosphatase/phosphodiesterase family member 5 (ENPP5); f) turripeptide; g) adenosine deaminase (ADA); h) ShK-containing protein; i) cysteine-rich secretory protein (CRISP); j) CAP-ShK protein; k) astacin metalloprotease; l) serine peptidase S1 (PS1) (Table 2).

**Table 2:** Feeding-related putative protein families. For each targeted protein family, the number of total and tissue-specific contigs, the range and median logFC (logarithm of Fold Change) of tissue-specific contigs and the inferred activities and mechanisms of action are reported

Protein family	Total contig number	SG contig number	SG logFC	MO contig number	MO logFC	Putative activities	Hypothesized mechanism
vWFA	177	24	6.1-16.2 (10.5)	0	0	antiplatelet	platelet scavenging
ACE	85	12	4.6-16 (10.3)	1	3.5	vasopressive	conversion of AngI to AngII
Porins	62	38	5.9-12.1 (8.8)	3	8.2-7.1 (7.1)	cytolytic	pore formation
ShK	42	25	5.7-13.5 (8.9)	0	0	anaesthetic	K+ channel blocking
PS1	41	2	11.8-14.5 (13.2)	0	0	anticoagulant	fibrino(geno)lytic, inhibition of coagulation factors
Meprin	33	8	5.1-13 (10.8)	1	7.6	anticoagulant; tissue degradation	fibrino(geno)lytic
Kunitz	27	1	5.1	8	6.4-10.1 (8.2)	anticoagulant	inhibition of thrombin and coagulation factors
CRISPs	26	12	7.0-10.6 (9.7)	0	0	anaesthetic; antiplatelet	ion channel blocking; radical superoxide scavenging
Turripeptide	17	0	0	1	8.5	anaesthetic	Gly channel blocking
ENPP5	17	4	7.3-10.8 (10.2)	0	0	antiplatelet	production of Ap4A
ADA	4	4	6.9-11.1 (8.9)	0	0	anaesthetic	removal of adenosine
CAP-ShK	3	3	8.8-10.2 (10.1)	0	0	anaesthetic	Ca2+ channel blocking

The salivary subset contains the highest number of protein classes (11) related to the venom, more than twice the number observed in the mid-esophageal subset (5), and a higher number of contigs for each class (Table 2 and Fig. 5a). Median logCPM values for each protein class, representing median reads abundance, are extremely variable, and are generally higher for the mid-esophageal transcripts (Fig. 5c). Tissue-specific enhancement, as measured by the logFC, is instead generally higher for salivary transcripts (Fig. 5b). The most overexpressed venom transcripts in the salivary glands both in terms of transcriptional enhancement with respect to the whole body (logFC) and in terms of relative abundance (logCPM) are ENPP5, ACE, CAP/ShK, vWFA and porins. Oesophageal most enhanced transcripts include Kunitz-domain proteins, turripeptide and meprins.

### Feeding-related protein classes

#### vWFA-like proteins

The most overexpressed salivary contig, comp107981_c1_seq2, carries three regions showing similarity to the vWFA domain, which is present in several proteins with different biological functions, including complement factors, collagens, integrins, ion channels, cochlin, human cartilage matrix protein, and others.

VWF is a huge multimeric protein that is an essential component of the coagulation cascade. VWF plays a key role in platelet-dependent primary homeostasis: it binds to exposed collagen after damage of blood vessels, then binds platelets initiating platelet plug formation. Moreover it binds coagulation factor VIII to protect it from degradation when in circulation [42, 43].

**Fig. 6:**
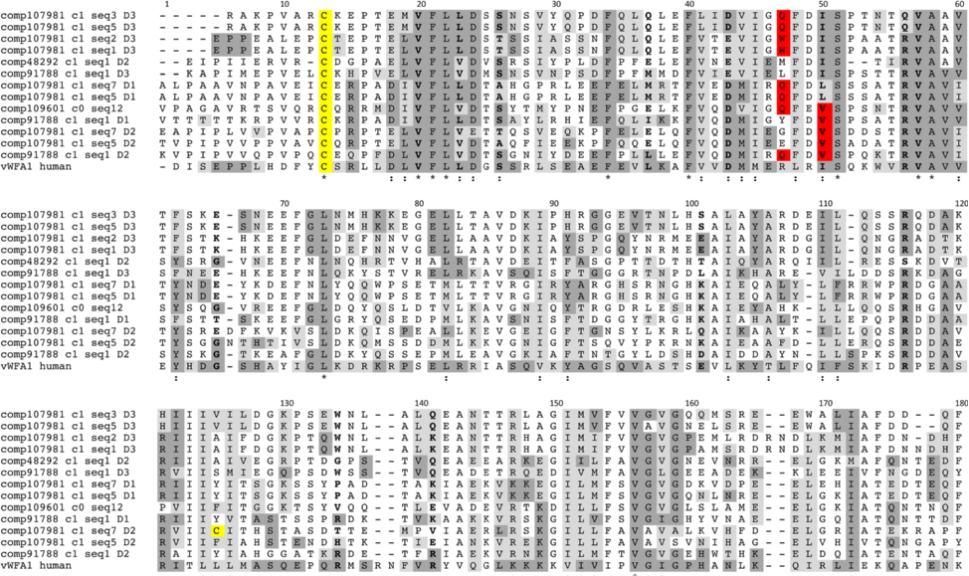
Alignment of the colubrarian vWFA domains with the human vWFA1. Cysteines in yellow, key residues for the interaction with platelet GpIb in bold, gain-of-function mutations in red. Identical and conserved residues with respect to human vWFA1 are highlighted respectively in grey and light grey and indicated respectively by asterisk and colon. Multiple identical copies of domains were excluded from the alignment

A total of 177 *Colubraria* contigs possess at least one vWFA domain; while none of them is overexpressed in the mid-esophagus, 24 contigs with one to three vWFA domains are markedly overexpressed in the salivary subset (Table 2; Additional file 3: Table S10). Most of the vWFA-like contigs possess a signal peptide and a stop codon, suggesting that they are complete and secreted proteins rather than fragments (Additional file 2: Figure S2). To better explore the similarities with human vWF domains, multidomain salivary proteins were split in domains that were aligned to human vWFA domains 1, 2 and 3 (Fig. 6). *Colubraria* vWFA-like domains are considerably similar (pairwise identity 17-23 %; pairwise similarity 35-35 %) to domain A1 of the human vWF, which is responsible for binding platelet GpIb [43, 44]. Therefore, by competing with the vWF of their prey, they may bind thrombocytes preventing their interaction with collagen and thus inhibiting thrombocyte aggregation, with a resulting anti-hemostatic action. The alignment of *Colubraria* vWFA-like domains with human vWFA1 domain highlights the conservation of most residues responsible for platelet binding (Fig. 6), according to molecular studies of several von Willebrand disease phenotypes (reviewed in [44]) and to molecular modeling of the interaction with platelets [45, 46]. Moreover, several residues in vWFA1 domain were found mutated in Type IIB von Willebrand disease, resulting in an increased affinity for platelets [44]; interestingly, two of such gain-of-function mutations were observed in some of the *Colubraria* contigs (R543W or R543Q: [45, 47]; I546V: [48]). This observation suggests that the affinity for fish thrombocytes may be higher in *Colubraria* vWFA-like proteins than in native fish vWFA1.

#### TFPI-like Kunitz-domain protease inhibitors

A total of 27 contigs possessing one or more Kunitz-domains were identified (Table 2). Only one of them, comp101225_c0_seq1, is enriched in both subsets (Table 2; Additional file 3: Table S11 and S12), while seven contigs (six of which are retrieved as isoforms of the same contig comp105558_c0_seq1-seq6), are enriched in the oesophageal subset (Table 2; Additional file 3: Table S12).

**Fig. 7:**
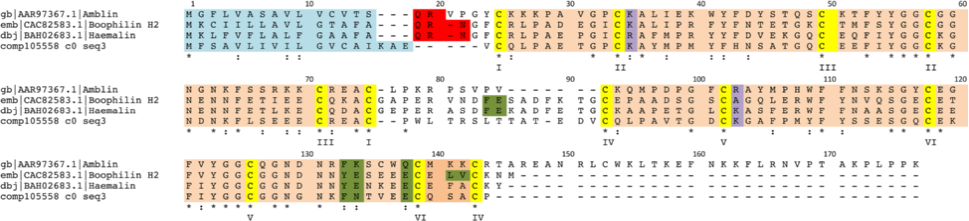
Alignment of two-Kunitz domains protease inhibitors. Predicted signal peptides in blue, Kunitz domains in light orange, conserved cysteines in yellow, residues binding Thrombin exosite in green, protease binding residues in purple, residues binding Thrombin active cleft in red. Identical and conserved residues are indicated respectively by asterisk and colon. Roman numbering indicate disulfide bonds pattern

The most complete contig, the 136-residues long comp105558_c0_seq3, highly overexpressed in MO, possesses two Kunitz domains separated by a short linker and displays high similarity values with hard ticks thrombin inhibitors, particularly with haemalin (pairwise identity 45 %, pairwise similarity 60 %), a recently described thrombin inhibitor from the bush tick *Haemaphysalis longicornis* [49] (Fig. 7). Signal P prediction identified a putative cleavage site between residues 15 and 16. The cysteine pattern is fully conserved between sequences and similar to what observed in TFPI, with 3 disulfide bonds predicted for each domain (Fig. 7).

**Fig. 8:**
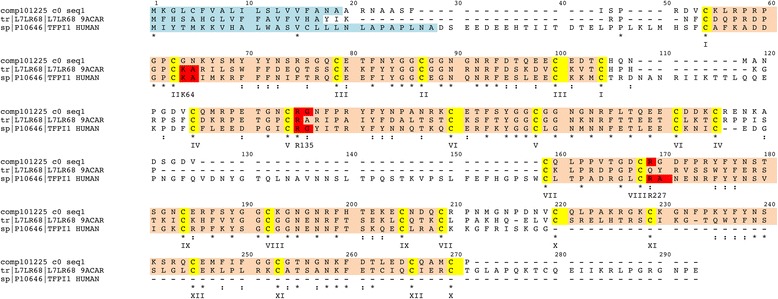
Alignment of the colubrarian four-Kunitz domain transcript with tick Tetralaris and human TFPI. Predicted signal peptides in blue, Kunitz domains in light orange, active sites in red. Identical and conserved residues are indicated respectively by asterisk and colon. Roman numbering indicate disulfide bonds pattern

A second contig, comp101225_c0_seq1, encoding for a putative protein of 167 residues with 4 Kunitz domains and a predicted signal peptide of 19 residues, is overexpressed in both tissues, especially in the mid-esophageal subset (Additional file 3: Table S11 and S12). This contig shows similarities with the Tetralaris, a 4 Kunitz-domains peptide isolated from the salivary gland of the Zebra tick *Rhipicephalus pulchellus* [50] (pairwise identity 33 %, pairwise similarity 39 %) and a high conservation level of key residues with human TFPI (although the latter possesses only three Kunitz domains) (Fig. 8).

A third contig, comp108520_c0_seq4 is remarkably long (1654 residues) and includes 6 Kunitz domains and 3 WAP domains. The WAP domain contains 8 characteristically spaced cysteine residues forming disulfide bonds. This multidomain contig is enriched in the MO subset (Additional file 3: Table S11).

Kunitz-like protease inhibitors have been found in the venom of a variety of venomous organisms, including snakes, cnidarians, wasps, spiders and scorpions [51], and are major components of the salivary secretions of blood-feeding arthropods [52]. In gastropods, they have been found in the venom gland secretion of *Conus* snails, where they were called ConKunitzins [53]. However, *Colubraria* Kunitz-like peptides are only distantly related with conKunitzin, being instead more similar to Kunitz peptides from hard ticks, especially the contig comp101225_c0_seq1 (the only Kunitz-like predicted peptide overexpressed in the salivary glands) that displays a high similarity with Tetralaris isolated in hard ticks. Intriguingly, multidomain Kunitz proteins are widely present in hard ticks (including the genera *Ixodes*, *Amblyomma*, and *Rhipicephalus*) but until now have not been detected in other species [52, 54]. A number of them exhibit a potent anticoagulant activity, often involving non-canonical bindings to inhibit specific coagulation factors, despite their overall similarity with TFPI [55].

Oesophagus produces different kinds of Kunitz-type TFPI-like protease inhibitor. Beside comp101225_c0_seq1, already described for the salivary subset, we detected the overexpression of a multidomain Kunitz/WAP (comp108520_c0_seq4) and a two Kunitz domain protease inhibitor (comp105558_c0_seq3). The latter is very similar to members of a hard-tick family of thrombin inhibitors that consist of pairs of Kunitz modules and include haemalin, amblin and boophilin [49, 55-57]. Although these molecules are only preliminarily characterized so far, a recent research highlighted their divergence from the antihemostatic factors identified in soft ticks (e.g. savigninin, monobin, ornithodorin) and evidenced their similarity to canonical Kunitz-type molecules such as BPTI [55]. The activity of haemalin, that is similar to contig comp105558_c0_seq3, was tested *in vitro* and resulted in the inhibition, even at low concentration, of plasma and fibrinogen clot, formation and platelet aggregation, indicating that haemalin is an anticoagulant for the common pathway of coagulation [49]. Boophilin, isolated from the whole body of *Rhipicephalus microplus*, is highly similar to haemalin, but it directly inhibits thrombin with a non-canonical mechanism, where a few N-terminal residues bind across the thrombin active-site cleft while C-terminal modules interact with the basic exosite I [57]. Amblin isolated from the haemolymph of *Amblyomma hebraeum* is quite different from other natural proteinase inhibitors, and its thrombin inhibitor activity still waits for an experimental confirmation [57, 58]. Although its activity remains to be tested *in vitro*, we can hypothesize that comp105558_c0_seq3 could act as a thrombin inhibitor in preventing blood clot formation to allow efficient feeding and digestion while passing through the oesophagus, as suggested for *H. longicornis* [49].

#### Adenosine deaminase (ADA)

We found 21 putative contigs showing similarity with adenosine deaminase (ADA), an enzyme that catalyzes the conversion of adenosine and 2’-deoxyadenosine to inosine and 2’-deoxyinosine [59] two of which are enriched in the salivary glands subset (Table 2; Additional file 3: Table S13).

These putative proteins display high levels of similarity with ADAs of other mollusk species (e.g. *Aplysia californica*) and hematophagous arthropods (including *Lutzomiya longipalpis, Phlebotomus dubosqui, Aedes albopictus, Culex quinquefasciatus* and *Anopheles gambiae)*. The alignment (Additional file 2: Figure S3) depicts the high levels of sequence similarity (up to 40 % pairwise identity and 58 % pairwise similarity) and the conservation of key residues for the enzymatic activity.

ADAs have been particularly studied in the salivary secretion of blood-feeding insects, namely *L. longipalpis* [60]*, C. quinquefasciatus* and *Ae. aegypti* [61], *Phlebotomus duboscqui* [62], *Chtenocephalides felii* [63] and *Glossina morsitans* [64]. In these species, the proposed activity for these molecules is the hydrolysis of adenosine, a molecule involved in pain perception [60, 65], thus reducing the perception of the parasite wound by the host. Additionally, the removal of adenosine produces inosine, a potent inhibitor of the production of inflammatory cytokines in vertebrates [61, 62]. Paradoxically, adenosine is also an immunosuppressive, vasodilatory and platelet aggregation inhibitor, and the presence of an enzyme that removes such a potentially useful molecule was explained in insects with the production of other more effective compounds counteracting these actions [60]. In *C. reticulata*, vasoconstriction apparently is not an issue, being instead advantageous to the parasite, while platelet aggregation can be counteracted by the action of ENPP5, Antigen-5 and vWFA-containing proteins.

#### Angiotensin-converting enzyme (ACE – M2)

We found a total of 85 contigs displaying similarity with single-domain angiotensin converting enzyme (ACE2) from mouse, and most interestingly with the only two known sequences identified in hematophagous animals (the duck leech *Theromyzon tessulatus* and the buffalo fly *Haematobia irritans*) (Table 2). ACE is a dipeptidyl carboxypeptidase belonging to the M2-metalloprotease family. Transcription of twelve contigs is enriched in the salivary glands (Additional file 3: Table S14) while a single putative contig enriched in mid oesophagous was retrieved (Additional file 3: Table S15). Partial contigs with a reduced length (less than 100 residues) and redundant sequences were excluded from the alignment that includes two *Colubraria* contigs overexpressed in the salivary subset, with a similarity to *T. tessulatus* and *H. irritans* ACEs up to 62 %, and a high level of conservation of residues in the active sites (Additional file 2: Figure S4). Both *Colubraria* contigs appear to be incomplete, with comp1091011_c0_seq4 having a predicted signal peptide but missing the C-terminal tail and comp109011_c1_seq2 aligning with the C-terminal end of the alignment.

In vertebrates, this zinc-dependant metalloprotease takes part in the renin-angiotensin system, converting the inactive decapeptide Ang I (angiotensin I) into the vasopressor octapeptide Ang II (angiotensin II). ACE also inactivates bradykinin, a vasodilator peptide, and thus contributes to blood pressure increase in mammals. In invertebrates, ACE-related genes have been cloned in a few insects [66-68], in the duck leech *Theromyzon tessulatus* [69], and recently ACE-encoding transcript was reported in the venom duct transcriptome of *Conus victoriae* [70], while the peptide was found in the proteomes of *C. purpuranscens* and *C. ermineus* [71]. For the cone snails it was suggested that the vasoconstrictory activity of ACE enzymes might play a role in envenomation, perhaps increasing the local concentration and effectiveness of the venom, or, more generally, interfering with cardiovascular homeostatic mechanisms as seen in other venomous animals. For a hematophagous feeder like *Colubraria,* ACE may play a role in the trophic physiology of the snail. In fact the proboscis in *Colubraria* is extremely thin and scarcely muscularized suggesting that it is not able to exert a strong suction [15]. Most likely, the passage of blood through the proboscis is due to the blood pressure of the fish, as confirmed by feeding observation (Oliverio & Modica, unpublished observations). In this context, increasing the blood pressure of the fish would maximize the blood income for *Colubraria*.

#### ShKT domain-containing proteins

Colubraria transcriptome contains 61 contigs with a ShKT domain (Table 2). ShK is a 35-residue peptide toxin originally isolated from the sea anemone *Stichodactyla helianthus* that acts as a potent inhibitor of voltage-gated and calcium-activated K channels [72]. The ShKT domain is short (36 to 42 amino acids) and characterized by six conserved cysteines forming three disulfide bonds [73]. ShKT domain is present in a large number of proteins, mostly metalloproteases, but also prolyl-4-hydroxylases, tyrosinases, peroxidases, oxidoreductases, and proteins containing epidermal growth factor-like domains, thrombospondin-type repeats, or trypsin-like serine protease domains.

**Fig. 9:**
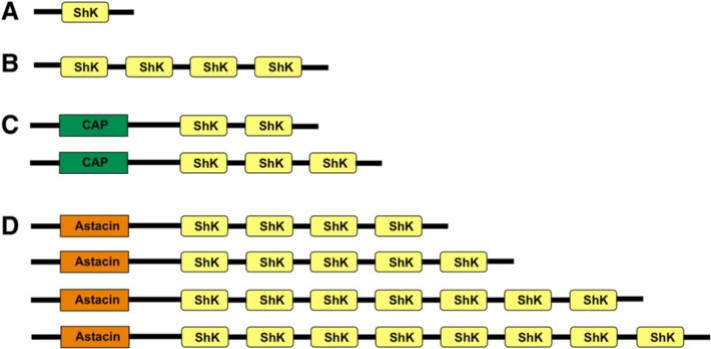
Organization of colubrarian ShKT domain-containing transcripts. **a**: single ShKT domain-containing transcript; **b**: four ShKT domains-containing transcript; **c**: CAP/ShKT domains-containing transcripts; and **d**: Astacin/multiple ShKT domains-containing transcripts

As for differential expression, 25 putative proteins containing ShKT domain were overexpressed exclusively in the SG subset, with high logFC and logCPM values (Table 2; Additional file 3: Table S16). At least 1 putative protein with a single ShKT domain, 5 putative proteins with 4 ShKT and respectively one with 5, one with 7 and one with 8 ShKT domains were found (Fig. 9).

The contig comp107594_c0_seq4 encodes for the single ShKT domain-containing putative protein that shows the highest similarity to the ShKT domain of sea anemones (pairwise similarity 41 %). This 99-residues putative protein is highly overexpressed in the SG subset and carries a 22 residues signal peptide as predicted by SignalP server (Additional file 2: Figure S5). Three contigs, comp103307_c0_seq1, comp105661_c0_seq2 and comp92668_c0_seq2, possess 4 ShKT domains, preceded by a short stretch displaying a partial and limited similarity with astacins. The absence of a putative signal sequence strongly suggests that they might be partial sequences (Additional file 2: Figure S6).

Five contigs contain a cluster of multiple ShKT domains downstream of a clearly identifiable and complete Astacin domain. They are similar to an Astacin-like Hydra metalloprotease with 3 ShKT domains, but the number of ShKT domains ranges from 4 to 8. In detail, three isoforms of the same contig, comp108799_c1_seq3, comp108799_c1_seq22, comp108799_c1_seq20, carry respectively 5, 7 and 8 ShKT domains (Fig. 9D; Additional file 2: Figure S7).

Three isoforms of the same contig (109272_c0_seq2, 109272_c0_seq3, 109272_c0_seq4) contain two or three ShKT domains preceded by a CAP domain, belonging to CRISP family (cysteine rich secretory protein). This structure shows high similarity to a 2-ShKT domain-containing protein from the parasitic nematode *Loa loa* and to snakes CRVPs (cysteine-rich venom proteins) (Fig. 9C). These isoforms are highly overexpressed in salivary subset (Table 2; Additional file 3: Table S17), but only in the sequence encoded by contig comp109272_c0_seq2 it was possible predicting a signal peptide (Additional file 2: Figure S8).

During *Colubraria* attack, the fish is anesthetized and usually does not react while the snail is feeding. However, after the detachment of the parasite the fish fully recovers without apparent damage. ShKT domain-containing proteins, which were found overexpressed in the snail salivary glands, are likely to take part in this reversible anesthetic action. ShK toxin is a potent inhibitor of voltage-gated and calcium-activated K^+^ channels. It has been hypothesized that, while ShK toxin *per se* is a potent blocker of ion channels in venomous animals, incorporation of the ShKT domain in other molecules (such as metalloproteases and serine proteases) may give rise to channel-modulatory enzymes [74]. K^+^ channels belong to the most abundant and diverse family of ion channels, and regulate a myriad of functions. Many venomous animal taxa have evolved potassium channel blockers, including cone snails and spiders [3, 51]. Several crucial differences may be highlighted in colubrarian transcripts with multiple ShKT domains: indeed, the number of cysteins may differ, as well as the conservation of key K/R residues, that have been considered crucial in the interaction with the K^+^ channel [73], may lack. However, the absence of these key residues in functionally active ShKT proteins was already described in snakes [75].

Interestingly, similar multidomain ShKT proteins have been recently identified in the venom of several species of the polychaete *Glycera* [75].

CAP proteins have been identified in the venom of a wide range of taxa, from Cnidaria to mammals [3, 51], and are particularly prominent in the venom of toxic reptiles [76]. The biological activity of these cysteine-rich proteins (CRISPs) has been subject of debate. As for snake CAPs, nevertheless, inhibition of smooth muscles contraction and of cyclic nucleotide-gated ion channels was reported [77]. *Colubraria* CAP-ShKT proteins appear related to snake CRVPs (cysteine-rich venom proteins) that may target voltage gated Ca^2+^ channels on smooth muscles [78].

Astacin metalloproteases play essential roles in diverse physiological mechanisms including digestion, early embryonic development, processing of the extracellular matrix and egg hatching. Astacins might also be good candidates to assist in the anticoagulant action, thanks to their fibrino(geno)lytic activity reported in spider venoms [79]. Astacin/ShKT-containing proteins of *Colubraria* could therefore both inhibit ion channels and contribute to impair hemostasis. Moreover, by degrading extracellular matrix molecules and basal lamina proteins, astacins might facilitate the spreading of the toxins in the body of the prey [79, 80] and inactivate several endogenous vasoactive peptides [81].

#### Meprin-like proteins

Beside the Astacin-ShKT containing proteins described above, in *Colubraria* transcriptome we found a total of 33 Meprin-like metalloproteases, comprising a contig encoding for an astacin domain followed by a MAM domain, overexpressed both in the salivary an in the oesophageal subset (Table 2). Among them, 8 contigs (all putative isoforms of the same putative protein encoded by comp108388_c4) are highly overexpressed in the salivary subset while a single contig (comp109603_c4_seq1) is overexpressed in the mid-oesophagus (Table 2; Additional file 3: Table S18, S19). These contigs show a pairwise similarity up to 50 % to Meprin A subunit B isolated from the bivalve *Crassostrea gigas* and a good level of conservation of active residues (Additional file 2: Figure S9).

Generally speaking, meprins are multimeric proteins, whose subunits are characterized by a unique combination of domains, namely Astacin, MAM (Meprin, A5 protein, and protein tyrosine phosphatase Mu), TRAF (tumor necrosis factor (TNF) receptor-associated factor), EGF (epidermal growth factor)-like domain, a transmembrane and a cytosolic domain [80]. Astacin is the catalytic domain responsible of the proteolytic activity, while MAM domain is involved in the homo-oligomerizations [82]. *Colubraria* contigs are devoid of most of the domains generally included in meprins architecture, possessing exclusively an astacin and a MAM domain. Despite the missing domains, the sequences obtained appear to be complete, with a signal peptide and a stop codon, suggesting that they may act as oligomeric astacins.

The presence of a MAM domain was detected to date in a number of other astacin-like proteins such as squid myosinases, HMP2 from Hydra and an enzyme from *Nematostella vectensis* [83-85]. Astacins as component of animal venom were described only in the brown spider *Loxosceles* venoms, where, beside a digestive role, it has been suggested that they could fulfill a fibrinogenolytic activity promoting anticoagulation of prey blood. It has also been hypothesized that astacins might contribute to venom spreading and inactivate prey vasoactive peptides [79-81]. Together astacins and meprins belong to the M12 family of metalloproteases that includes important components of snake venoms [86]. In Viperidae, metalloproteases M12 are the most abundant venom toxins [87, 88], and are involved in a variety of physiological activities including hemorrhagic and fibrinolytic effects [89].

#### Cysteine-Rich Secretory Proteins (CRISPs)

**Fig. 10:**
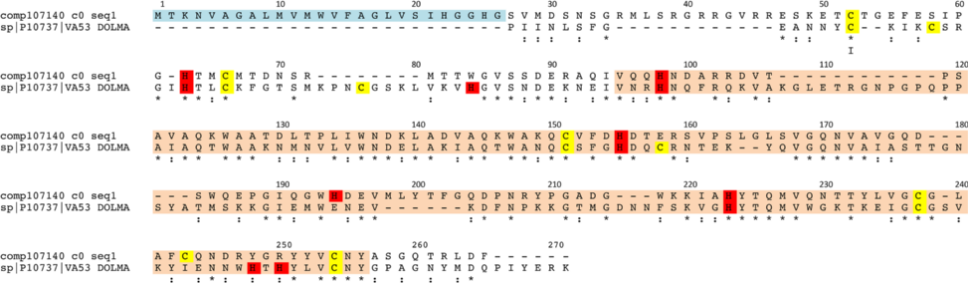
Alignment of the colubrarian putative Ant5 with *Dolichovespula maculata* Ant5. Predicted signal peptide in blue, SCP-domain in light orange, Histidine residues in red, Cysteine residues in yellow. Identical and conserved residues are indicated respectively by asterisk and colon

Beside the CAP-ShKT group, 25 other contigs with homology to CRISPs (Table 2) were found in *Colubraria* transcriptome, 12 of which are overexpressed in the salivary subset (Table 2; Additional file 3: Table S20), a number that elevates to 15 if we include the CAP-ShKT contigs described above (Additional file 3: Table S17). Among the most enriched in the salivary subset (logFC: 10.6; logCPM: 8.1; SI: 65 %), contig comp107140_c0_seq1 possesses a predicted signal peptide of 26 residues and aligns well with venom allergen 5, a major constituent of vespid venom. It also displays a certain degree of similarity with homologs isolated from the hematophagous arthropods *Dipetalogaster maximus* and *Triatoma infestans* [90, 91] (pairwise identity 21 % and 19 %, pairwise similarity 30 %), including the conservation of crucial Histidine residues (Fig. 10). Contig comp107140_c0_seq1 is shorter than its arthropods counterparts, suggesting that the sequence might be incomplete. Antigen-5 family comprises a considerable number of members, whose biological functions are mostly unknown. Some of them have been recruited to accomplish roles in blood feeding during the evolution of hematophagous taxa, and have been identified in most of the hematophagous arthropods studied to date where they can fulfill different tasks. Inhibition of collagen-induced platelet aggregation has been recently reported for antigen-5 of the hematophagous Triatomine bugs *Dipetalogaster maximus* and *Triatoma infestans*, the vectors of Chagas disease [90, 91]. A recent study revealed that these proteins are Cu^2+^ antioxidant enzymes that are able to inhibit collagen-induced platelet aggregation, ATP secretion and thromboxane-A production by a unique mechanism involving the removal of radical superoxide [92]. Moreover, scavenging of radical superoxide down-regulates a myriad of pro-inflammatory reaction, as it limits the production of inflammatory extracellular ROS by neutrophils, platelets and other cell types [92]. It can be hypothesized that antigen 5 protein could have the same activity in *Colubraria,* to reinforce anti-platelet-aggregation capabilities of the snail.

The other contigs encoding for CRISPs appear to be largely incomplete, but despite being quite different in length, they are generally similar to Mr30 and Tex31 proteins isolated in two *Conus* species, *C. marmoreus* and *C. textile* [93, 94] (Additional file 2: Figure S10). Although the biological function of these proteins still needs to be clarified, an inhibitory activity on ion channels was hypothesized for them [93, 94].

#### Peptidases S1

A total number of 41 contigs similar to Peptidase S1 were found (Table 2), only 2 of which are differentially expressed and enhanced in the salivary subset (Table 2; Additional file 3: Table S21). These putative proteins are quite different: comp108333_c0_seq1 appears to be a chymotrypsin, while comp98826_c0_seq1 is more similar to trypsin-like peptidases isolated in cephalopods [95] and the fibrinolytic enzyme of the Echiurid worm *Urechis unicinctus.* The trypsin-like comp98826_c0_seq1 does not possess a predicted signal peptide, thus might be incomplete, and displays only a partial level of conservation of key residues at both the C-terminal active site and the substrate binding site (Additional file 2: Figure S11).

The other contig encoding for a putative peptidase S1, comp108333_c0_seq1, aligns with chymotrypsin of the ant *Harpegnatos saltator*, the fly *Glossina morsitans* and with a secreted salivary protein of the tick *Ixodes scapularis*. However, the residues corresponding to the active sites of the catalytic triad are not conserved, raising doubts on its effective putative activity (Additional file 2: Figure S12).

The peptidase S1 (PS1) family of serine proteases is a large peptidase family, including chimotrypsin, trypsin and elastase activities. Biological roles of the PS1 are extremely diverse, ranging from digestion to immune responses. The venom of several taxa of both vertebrates and invertebrates contains PS1 that are the dominant venom component in several species of cephalopods, in male platypuses, and in Remipedia [51, 96]. Activities of PS1 in the venom are diverse and are involved in pain, inflammation, smooth muscle contraction, vasodilation and prevention of blood clotting. In *Colubraria*, members of PS1 family constitute only a reduced fraction of the salivary secretion, but include two well-differentiated putative proteins that may participate to the overall envenomation process, possibly exploiting an anticoagulant action. These proteins are the trypsin-like protein similar to cephalopods peptidases S1 and to echiurid fibrinolytic enzyme, and the chymotrypsin-like peptidase that is devoid of the conventional catalytic triad. Recent research identified in snake venoms a number of PS1 with unconventional catalytic triads that show activity towards a wide array of proteins involved in hemostasis, being able to degrade fibrinogen, fibrin, prothrombin, factor X and plasminogen [97].

#### Ectonucleotide pyrophosphatase/phosphodiesterase family 5 (ENPP5)

A total number of 17 contigs encode for members of the ENPP5 family, which hydrolyze 5’-phosphodiester bonds of nucleotides and their derivatives, resulting in the release of 5’-nucleotide monophosphates (Table 2). Four slightly different isoforms of a same contig are overexpressed in the salivary glands (Table 2; Additional file 3: Table S22) and encode for putative proteins of 441 residues long (except for comp109445_c0_seq3, that carries a gap of 82 residues), with a signal peptide of 26 residues, and highly similar (up to 44.3 % identity) to ENPP5 enzyme from *Crassostrea gigas*. Molluscan sequences lack the transmembrane region that is instead present in human ENPP5 (Additional file 2: Figure S13).

ENPP5 family is a versatile group of enzymes with a broad substrate specificity [98, 99]. It was shown that members of the ENPP5 family are capable of hydrolyzing dinucleoside polyphosphates, a group of nucleotides that recently has attracted considerable interest because its members act as extracellular signaling molecules in a broad variety of tissues [99]. It was also shown that they are involved in platelet aggregation, with a potent inhibitory effect reported for diadenosine 5’,5”’-P^1^,P^4^-tetraphosphate (Ap_4_A) [100]. Ap4A can be produced by ENPP5 hydrolysing Ap5A, suggesting an indirect anti-aggregant activity for *Colubraria* ENPP5, as indicated by the overexpression in the salivary glands.

#### Porins

A total of 62 contigs were described in the transcriptome, showing sequence similarity to echotoxins and conoporins, pore-forming lectins with lethal and haemolytic effects described respectively in the caenogastropod *Monoplex echo* and in the Conoidean *Conus consors* [101, 102] (Table 2). These proteins belong to the sea-anemones cytolysine family of Actinoporins [103, 104], whose members are able to form pores in the cell membrane.

Porin contigs are mostly represented in the salivary subset that contains 38 overexpressed contigs (Table 2), one of which is shared with the MO subset (but with a higher enhancement in the SG subset; Additional file 3: Table S23). Two contigs, comp89661_c0_seq1 and comp93847_c1_seq1, are specific of the MO subset (Table 2; Additional file 3: Table S24).

Alignment of the 8 most complete contigs (length between 192 and 268 residues and signal peptide predicted) with echotoxins and conoporins is displayed in supplementary Additional file 2: Figure S14; noticeably, residues important for the interaction with the membrane are not completely conserved [105]. Overall, *Colubraria* porins display the highest similarity to conoporin and to echotoxin A (pairwise identity values ranging respectively from 27 % to 41 % and from 25 % to 33 %; pairwise similarity from 40 % to 53 % and from 40 % to 51 %).

Noteworthy, the mid-esophageal contig comp89661_c0_seq1 displays the highest similarity to conoporin, a finding that might be related to the common ontogenetic origin of the mid-oesophagous of *C. reticulata* and the venom duct of Conoidea.

Porins are able to perforate cellular membranes in a multistep process. Such process involves i) recognition of membrane sphingomyelin using aromatic rich region and adjacent phosphocholine (POC) binding site, ii) firm binding to the membrane (mainly driven by hydrophobic interactions) accompanied by the transfer of the N-terminal region to the lipid-water interface and iii) eventually, pore formation after oligomerization of several monomers [106]. Echotoxins, the first protein toxins isolated from marine gastropods, differ from actinoporins in having affinity for gangliosides instead of sphingomyelin [101, 105]. For some of the gastropod echotoxins identified so far, a cardiac stimulation activity has been reported. The high expression levels of porins in the salivary glands of *C. reticulata* can be related to the need to access blood vessels.

#### Turripeptide

A single contig, comp109534_c9_seq2, enhanced in the MO subset (Table 2), displays a high level of similarity (pairwise identity 55.7 %, pairwise similarity 69.5 %) with known turripeptides, from *Lophiotoma olangoensis, Iotyrris cingulifera*, and *Gemmula speciosa* (Additional file 3: Table S25; Additional file 2: Figure S15) [107]. These are conotoxin-like peptides discovered in the venom gland of the Conoidean family Turridae, characterized by three disulfide bonds arranged according to the IX cysteine framework. This pattern is typical of the poorly known P superfamily of conotoxins, whose sequences are however quite divergent when compared to turripeptides [108].

Contig comp109534_c9_seq2 encodes for a 72 residues peptide, with a 20 aminoacids predicted signal peptide, and possibly a 5 residues propeptide region, according to similarity with known turripeptides (Additional file 2: Figure S15).

Turritoxins are reported to act as neurotoxins by inhibiting ion channels, and possibly also as serine protease inhibitors, given that they posses the Kazal serine protease inhibitor signature. Their 6-Cys pattern was first characterized in the peptide BeTXIIa from the venom of the vermivorous species *Conus betulinus* [109], and subsequently found in a few “spasmodic peptides”, that define the P-superfamily of conotoxins and the Cys framework IX [110]. Framework-IX venom peptides have additionally been found in members of families Turridae and Terebridae [111, 112], however the sequence similarity to peptides of Conidae P-superfamily is generally low [108].

An analysis of feeding habits of conideans reveals that framework IX peptides are not produced by fish-hunting cone snails but they are expressed in the venom duct of molluscivorous cone snails and vermivorous conoideans (including also Terebridae and Turridae beside some species of cone snails) [108]. The molecular target of any framework IX-conopeptide has not been identified thus far. However, experimental evidences suggest, at least for some members of the P-subfamily, an action on glycine receptors [110], ligand-gated ion channels belonging to the nicotinic acetylcholine receptor family, which are important targets for neuroactive drugs [113]. Gly receptors are in fact widely distributed inhibitory receptors, that are involved in the regulation of the motor circuits of the spinal cord and have inhibitory synapses in afferent sensory neurons, including pain fibers [113, 114], being ideal targets for peripheral anesthetic compounds. The oesophageal overexpression of turritoxin-like contig comp109534_c9_seq2 is in agreement with the structural homology of Conoidean venom duct with the colubrarian mid-oesophagus [26]. The expression of other families of conopeptides in salivary glands was reported for *Conus pulicarius* [115], but no conopeptides were found in a recent transcriptome of salivary glands of *C. geographus* [20]. Expression of turritoxins does not appear to be restricted to gastropods, as it was also recently detected in the venom of the polychaete *Glycera* [75].

## Conclusion

We present here the first molecular characterization of the alimentary secretion of a non-conoidean neogastropod, by deciphering secretory repertoires of salivary glands and mid-oesophagus of a hematophagous snail.

### Physiology of feeding

The biochemical characteristics of the feeding-related classes of molecules identified in the *Colubraria* transcriptome allow to hypothesize their functional role and the physiology of the glandular tissues in the light of *Colubraria* feeding habits. The transcriptomic profiles of the two secretory tissues studied are different, with a reduced overlap in the classes of molecules dominating their secretions, and indicate a large degree of specialization, with a significantly higher complexity in the salivary glands. Anticoagulant activities in the saliva, which is secreted both into the prey and along the extremely long anterior oesophagus, can be ascribed to the synergic action of several molecules, with a crucial role possibly played by the vWFA domain-containing proteins, encoded by numerous contigs overexpressed at the highest levels in the salivary subset. The mid-oesophageal transcriptome is apparently less complex than the salivary one and is dominated by transcripts encoding for a turritoxin-like protein and for a two Kunitz-domains protease inhibitor

These expression patterns reflect the different physiological roles of the two tissues. We hypothesize that the mid-oesophageal turritoxin is produced when the snail approaches the fish and, as in the case of the venomous cocktail of *Conus geographus*, it may be involved in the induction of a “hypnotic” state in the fish. *C. geographus*, as other so called net-hunter cone snails, engulfs fish (or even a small school of fishes) with its highly extensible rostrum before stinging. Apparently, when the fish is approached by a cone snail, it remains in an almost hypnotic state until the snail is close enough to engulf it [22] displaying a behavior extremely similar to *Colubraria* predatory activity. The biochemistry of the induction of “hypnosis” has not been investigated to date in *Conus*. In *Colubraria* the use of an oesophageal secretion in the external environment could be possible due to the loss of the valve of Leiblein, a structure that in neogastropod separates the mid from the anterior oesophagus and thus from the environment [15, 26].

Afterwards, when the blood meal is ingested, the main task of the oesophagus would be avoiding blood clotting during its passage through the stomach, where it will be digested. Such a physiological role may be sustained by the haemalin-like two-Kunitz domain protease inhibitor. Haemalin is a thrombin inhibitor, produced in the oesophagus of the bush tick *Haemaphysalis longicornis*, able to avoid clotting of the blood meal until digestion is completed [49].

The salivary glands, that discharge their secretion at the tip of the proboscis, very close to the external environment, produce a more complex venom probably in response to their more diversified tasks. First, when the proboscis contacts the fish, the snail has to gain access to the blood. The massive secretion of porins, maybe assisted by the metalloproteases, probably elicit a potent cytolytic activity [79, 106], that associated with the scraping action of the radula allows the snail to open large circular wounds on the fish skin (Fig. 2B). Unquestionably, an anesthetic activity is crucial to efficiently accomplish blood-feeding in any hematophagous taxa. In *Colubraria* this anesthetic activity is likely due to the concerted activity of ShKT-containing proteins, especially the CAP-ShKT that act as K channel modulators [78], and ADA, that may reduce local pain perception by removing adenosine [60]. The anticoagulant action at the site of the wound and in the anterior digestive tract is mediated by the salivary production of a complex mixture of anticoagulant compounds. The vWFA-containing proteins, novel proteins here described for the first time, may act as platelet anti-aggregants and target primary hemostasis, throughout a mechanism still to be defined but probably including direct scavenging of thrombocytes. Anti-platelet activity can be assisted by the Antigen-5 protein, via a radical superoxide removal mechanism [92]. ENPP5 can participate to disarrange primary hemostasis, producing Ap4A that reinforce the inhibition of thrombocyte aggregation [100]. Secondary hemostasis may be targeted by the 4 Kunitz domain protein, similar to the tick *Rhipicephalus pulchellus* Tetralaris, a potent thrombin and TF/FVIIa complex inhibitor [52, 54], and to TFPI, that modulates factor Xa. Astacins and peptidases S1 also likely contribute to impair hemostasis, as described e.g. in snakes and spiders, due to the fibrinogenolytic activity of astacins [79], and to the hemorrhagic–anticoagulant activity of PS1 that involves the degradation of several hemostatic factors [97]. Finally, salivary glands produce ACE that owing to its vasopressive activity can increase cardiac frequency in the fish and maximize blood income through the snail foregut, considerably reducing *Colubraria* feeding time. Vasoconstriction is a defense mechanism that is part of the hemostatic system, and is generally counteracted by hematophagous animals; interestingly, in a “passive feeder” such as *Colubraria* it becomes advantageous to the parasite, explaining the recruitment of ACE enzyme in the venomous cocktail.

### Evolutionary considerations

Most hematophagous animals produce a complex mixture of anti hemostatic molecules that act synergistically to impair hemostasis in the host, the natural response to vascular injury aimed to arrest bleeding and based on the triad of platelet aggregation, blood clotting and vasoconstriction. While *Colubraria* secretion does not block vasoconstriction, which is advantageous to this parasite, it counteracts both primary and secondary hemostasis. Primary hemostasis is targeted by several molecules produced in different hematophagous species, that act at various stages, including salivary apyrases, epinephrine and serotonine binding molecules and nitrophorins in several arthropods; lipocalins in *Rhodnius prolixus*, triatomines and ticks; collagen binding proteins as antiplatelets in mosquitos and leeches; integrin antagonists in ticks and hookworms [6]. Inhibitors of secondary hemostasis are extremely variable as well, in their molecular masses, targets and inhibitory mechanisms, with thrombin and FXa being the most common targets [5, 7].

Apparently, while inhibition of secondary hemostasis in *Colubraria* involves mechanisms already described in other animal groups, this snail evolved a unique molecular arsenal to impair primary hemostasis, as a key role would be played by vWFA-like proteins, assisted by ENPP5 and Antigen-5. The evolution of such arsenal could have been triggered by the high efficiency of the coagulation system in fishes that need to rapidly repair vascular damage, especially in the gills, where blood supply is maximum and very close to the external environment. In fact, although hemostasis in fish involves the same mechanisms and factors described in mammals (except for the presence in fish of nucleated thrombocytes instead of anucleated platelets), coagulation time is generally shorter, due to the high levels of coagulation factors contributing to high activity of the extrinsic and intrinsic pathway [116]. Further molecular and biochemical studies on the anticoagulant secretions of other hematophagous parasites of fishes, such as fish lice and lampreys, could elucidate mechanisms of evolutionary “arms race” between fishes and their parasites.

Surprisingly, we detected a very low level of similarity between *Colubraria* and *Conus* biochemical predatory arsenals, restricted to a single molecule, the turripeptide. Additionally, such molecules are not exclusive of gastropods as demonstrated by their recent discovery in the venom gland of the polychaete *Glycera* [75]. On the other hand, the presence in *Colubraria* of multiple Kunitz-domains proteins that were found to date only in hard ticks (Ixodidae) [52] indicate some degree of physiological convergence between colubrariids and ixodid ticks. It should be remarked, for example, that both hard ticks and *Colubraria* share a feeding behavior that involves a reversible attachment to the host that can last several hours [117].

Given the close phylogenetic relationship of Mollusca and Annelida [118], we expected to find strong affinities between the biochemical arsenal of *C. reticulata* and that of leeches and of the recently investigated polychaete *Glycera*. Again, very limited similarities were instead detected, namely the presence in *Glycera* of porins, turripeptide-like, CAP/ShKT and Astacin/ShKT [75] and the similarity of leech ACE to its *Colubraria* counterpart. This indicates a different evolutionary pathway for the onset of hematophagy arsenal in the two lineages, and a dominant influence of convergence between distantly related but functionally similar taxa, such as *Colubraria* and hard ticks. Moreover, the presence of anesthetic multidomain ShKT proteins is shared with lower metazoans such as Cnidaria, suggesting that they might represent a basal animal toxin arsenal.

More tailored investigations on the main feeding-related protein classes here identified are needed to shed light on their activities, and also to define proteins involved in other biological roles such as the modulation of the inflammatory response of the host. However, our results undoubtedly indicate that marine predatory gastropods could be a valuable study target to identify novel bioactive molecules. Conoidea investigated to date constitute only a fraction of the overall Neogastropoda trophic diversity, suggesting that this animal group as a whole is a plentiful reservoir of bioactive peptides worth of detailed investigations.

## Methods

### Samples and tissues collection

A total of 30 specimens of *Colubraria reticulata* were collected alive at Sidi Jmour (Djerba Is., Tunisia: 7-12.vi.2013; 33°49.5’N, 010°44.5’E), in shallow waters (0.3-2.5 m depth), under rocks and boulders. Specimens were kept alive in aquarium until sample preparation. Twenty-four specimens were dissected on ice, and target tissues (mid-oesophagous and salivary glands; hereafter MO and SG) were collected and used to prepare three tissue-specific biological replicates constituted of 8 samples each (SG 1-3 and MO 1-3). Additionally, 6 animals were used to prepare three biological replicates of whole-body reference, made up of 2 samples each (WB 1-3). During preparation of the whole-body samples, where we encountered engorged snails, the stomach full of blood was excluded from the sample, to avoid contamination with fish transcripts.

Samples were preserved in RNA later at -80 °C until RNA extraction.

### RNA extraction, library preparation and next generation sequencing

Total RNA from each sample was isolated using a QiagenRNeasy Mini Kit according to manufacturer’s instructions. FastPrep-24 homogenizer (MP Biomedicals) was used to process approximately 30 μg of each sample (30 sec at 4.0 m/s). RNA quality and quantity was assessed using a Bioanalyzer 2100 (Agilent Technologies, Palo Alto, USA) and a Qubit v2.0 fluorometer (Life Technologies, Darmstadt, Germany), respectively.

For each sample, 250 ng of high-quality RNA (RIN value > 8) was used to construct nine barcoded sequencing libraries with the Illumina TruSeq RNA sample preparation kit (Low-Throughput protocol) according to the manufacturer’s instructions (Illumina, San Diego, USA). To increase the average library insert size, input RNA was chemical fragmented at 94 °C for 1 min.

The nine barcoded samples were equimolar-pooled and clustered template cDNA was sequenced in an Illumina HiSeq2000 platform with 209 cycles (101 cycles for each paired-read and seven cycles for the barcode sequences).

### Bioinformatics analyses and differential expression

After sequencing we obtained 329,576,088 raw reads (from 33,213,610 to 38,909,488 reads per sample), each 101 bp in length that were quality-controlled (quality score higher than Q36 where Q40 is the maximum value) before assembly, read mapping and downstream analyses. First, SeqPrep (https://github.com/jstjohn/SeqPrep) was used to remove the remaining adapters and to merge the overlapping paired-reads. The reads were then trimmed by quality in CLC Genomics Workbench v6.5 (CLC bio, Aarhus, Denmark). Low-quality reads (CLC parameter ‘limit’ set to 0.02) and reads shorter than 50 nucleotides were excluded. Finally, a custom database constituted by bacteria, fungi, virus and protozoa (source: NCBI Reference Sequence, RefSeq, March 2014) was used as reference to align the reads of each sample. Reads that aligned to these potential source of contamination were removed. A final dataset of 220,305,266 filtered reads (140,615,306 paired reads, mean length 99 bp, and 79,689,960 single merged reads, mean length 154 bp) ranging from 17,993,689 to 27,248,309 reads per sample was used to build the transcriptome assembly.

The filtered reads were *de novo* assembled using Trinity v20140717 [119]. Trinity was run on the two sets of paired-end and single-end (merged) sequences with the fixed default k-mer size of 25 and a minimum contig length of 60 bp. To reduce the number of isoforms, the Pasa assembly algorithm implemented in the Butterfly module of Trinity was used. The produced assembly was subjected to similarity search against three different databases: a) NCBI’s non-redundant (NR) database; b) EMBL’s UniProtKB/Swiss-Prot; c) a custom nucleotide database including the transcriptomes of three mollusc species *Ilyanassa obsoleta* (PRJNA79721), *Nucella lapillus* (PRJNA217409), *Conus geographus* (venom duct transcriptome; PRJNA167726), plus the salivary glands EST library of the leech *Macrobdella decora* (LIBEST_028114). BLASTx (NR and Swiss-Prot databases) and BLASTn (mollusc databases) algorithms [120] with a cut-off e-value of 10^−6^ were used for similarity searches. The assembly was further scanned for the presence of functional protein domains using InterProScan [28] with the following search methods: SignalP, TMHMM, Pfam, SMART, Tigr, ProfileScan. Transcripts that aligned to proteins or transcripts contained in the databases and/or had InterPro match were retained for downstream analyses.

The obtained final set of sequences (144,380 transcripts) was used as reference for read mapping and differential expression (DE) analyses. Read mapping for each sample was performed using Bowtie2.2.3 [121] and gene expression level for each transcript was estimated by RSEM v1.2.15 [122]. The read-count table was exported and DE analysis was carried out using the Bioconductor edgeR v2.14 [123] R v3.1.0 package.

Three different differential expression (DE) analyses were carried out comparing the three different tissues (WB, MO and SG) in a pairwise manner. False discovery rate (FDR) was applied to correct p-values generated by edgeR’s exact test (transcripts showing FDR < 0.05 were considered DE). Following a conservative approach, we considered enriched those DE contigs having logFC values (the log_2_ fold change between tissue-specific vs. whole body relative abundances) of 3 or higher.

Functional annotation of the DE transcripts was carried out by Blast2GO [124], setting the NCBI’s NR as reference database (E-value < e^−6^, annotation cut-off > 55, GO weight > 5). Enrichment analyses, using the Fisher’s exact test implemented in Blast2GO, were applied to identify significantly over-represented gene ontology (GO) terms in the DE transcripts (test sets) when compared to the whole assembly (baseline set).

The open reading frame (ORF) for each transcript was detected using two methods: a) ORF were predicted using the TransDecoder module implemented in the Trinity package, and the longest ORF among those matching the same protein was selected; b) ORF was detected, for each transcript, using the output coordinates of the BLASTx similarity search against NR or Swiss-Prot (when no BLAST hit to NR was recorded).

Further inspection, to identify peptides potentially involved in feeding was carried out with user-defined scripts in R applying two strategies.

First, the whole transcriptome was screened out for contigs with NR hits to known proteins with potential neurotoxic and hemotoxic activity, according to previously published reference data [3, 51]. Similarity with the following protein classes was investigated: a) conopeptides; b) protease inhibitors; c) peptidases S1; d) phospholipases A2; e) lectins; f) metalloproteases; g) cysteine-rich secretory proteins (CRISPs); h) apyrases.

Second, particular attention was given to the contigs (even with no NR hits) that are enriched over a specificity threshold in each tissue. We defined a specificity index (SI) for each contig in the two tissue-specific subsets, normalizing the logFC of each contig to the logFC of the most overexpressed contig in each tissue. Contigs with a SI > 65 % were singularly screened to identify potential feeding-related activity in both tissues, and manually annotated.

Alignments for the main protein classes were built using ClustalW [125].

Salivary (935 contigs), oesophageal (184 contigs) and whole body (1048 contigs) catalogues were also manually translated using Virtual Ribosome - version 1.1 server [126] and only peptides starting with Met were considered. The probability to encode for a signal peptide was evaluated using two on-line prediction servers (SignalP and PrediSi) [37, 38].

### Ethics statement

All experiments were conducted in accordance within Italian laws, and thus required no ethics approval for the animals used in the study. Field permits are not required for this species.

### Availability of supporting data

The short read DNA sequences and the de novo transcriptome assembly have been deposited in the European Nucleotide Archive (ENA) under the accession code PRJEB9058.

## Additional files

Additional file 1: Tables S1-S9. Tables describing general transcriptome features and tissue-specific differential expression, including enriched PFAM protein families. Additional file 2: Figures S1-S15. Tissue-specific histogram of enriched PFAM families; alignments of the putatively feeding-related colubrarian sequences. Additional file 3: Tables S10-S25. Tables describing tissue-specific expression values for each feeding-related protein class.
