# Supplementary material for: The venomous cocktail of the vampire snail Colubraria reticulata (Mollusca, Gastropoda)
Source: BMC Genomics. 2015 Jun 9;16(1):441. doi: 10.1186/s12864-015-1648-4 (PMC4460706; doi:10.1186/s12864-015-1648-4)
Supplement: Additional file 2: Figures S1-S15. — Tissue-specific histogram of enriched PFAM families; alignments of the putatively feeding-related colubrarian sequences. [file 12864_2015_1648_MOESM2_ESM.pdf]

Additional file 2 – Figure captions

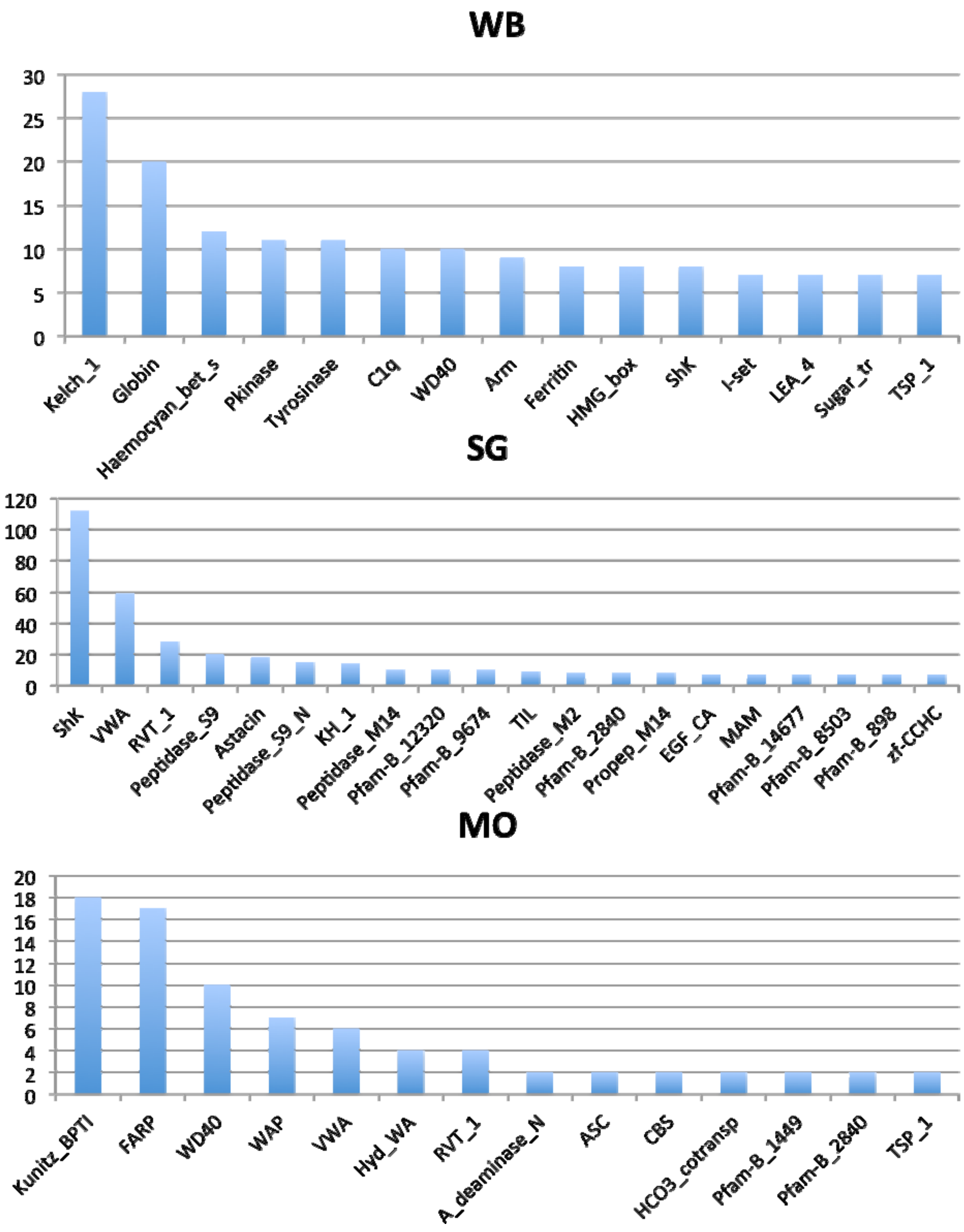

Figure S1. Number of occurences of the most enriched gene families in the whole body and in each tissue. For each subset, the frequency of the most represented PFAM gene families was reported

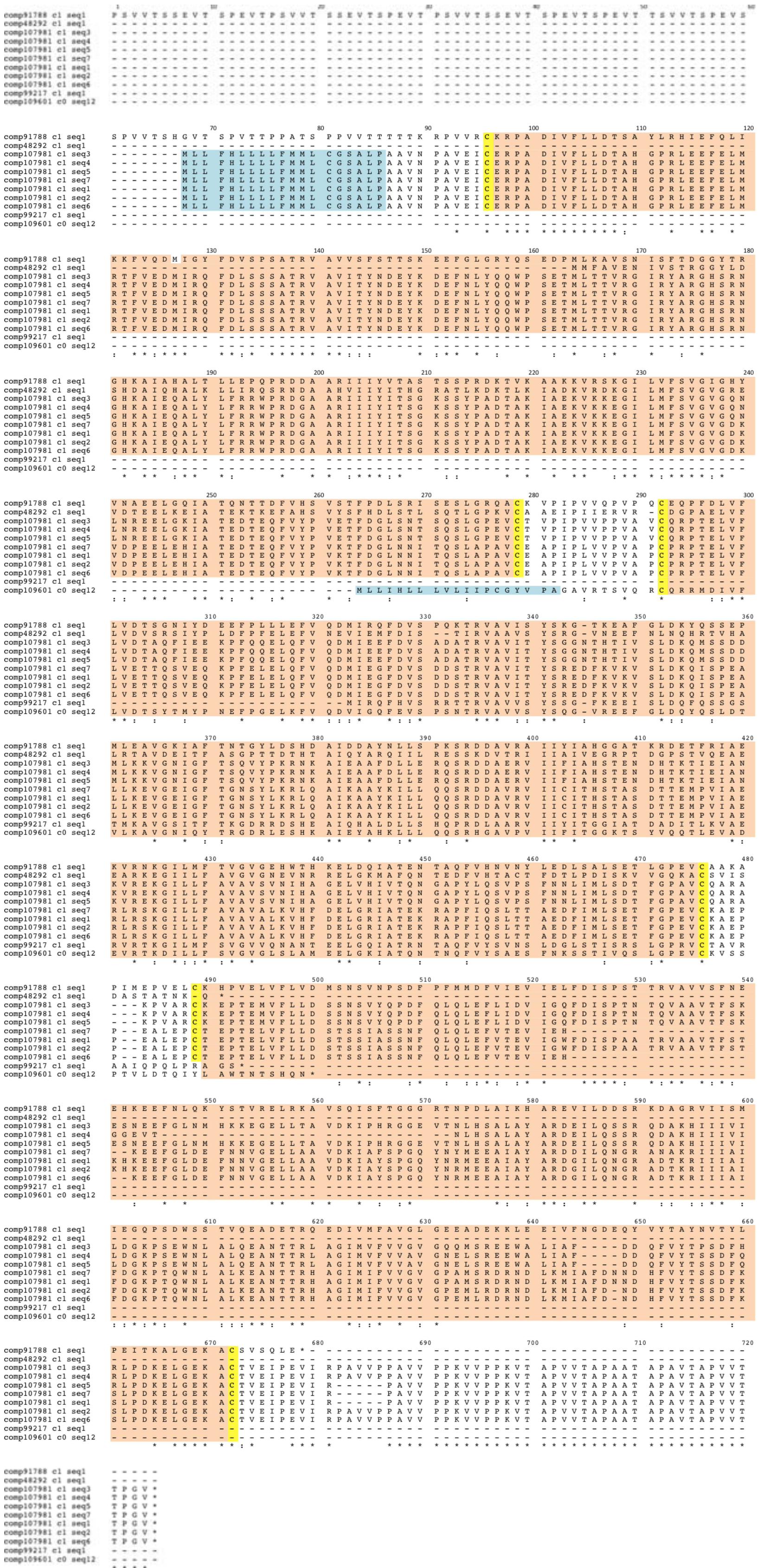

Figure S2: Alignment of the colubrarian vWFA containing contigs. Signal peptides in blue, vWFA domains in orange, Cysteines in yellow. Identical and conserved residues are indicated respectively by asterisk and colon.

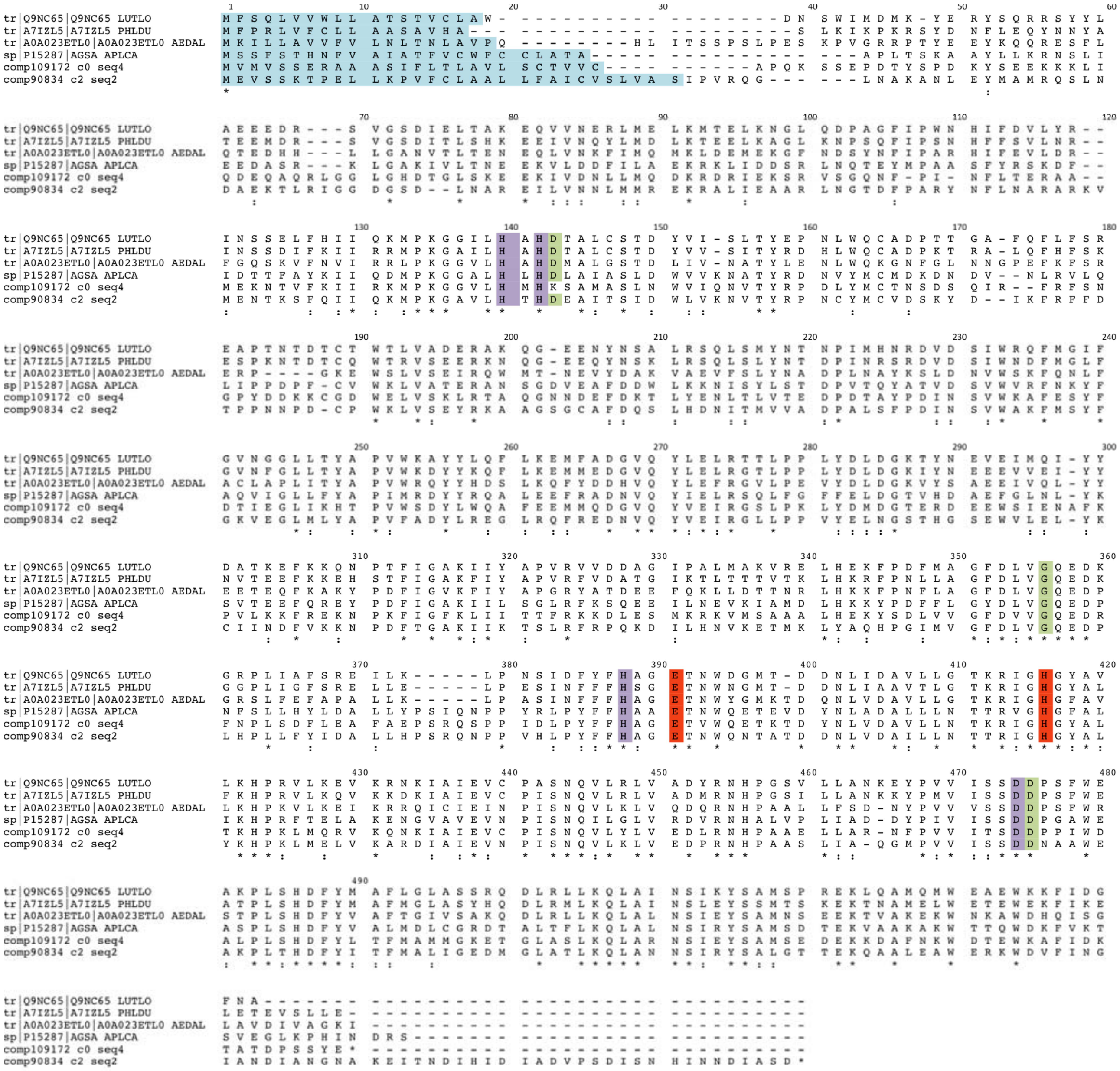

Figure S3: Alignment of the colubrarian ADA contigs with ADA from three hematophagous arthropods (*Lutzomyia longipalpis, Phlebotomus dubosqui, Aedes albopictus*) and the mollusk *Aplysia californica*. Signal peptides in blue, substrate-binding sites in green, zinc-binding catalytic sites in purple and active sites (proton acceptors/donors) in red. Identical and conserved residues are indicated respectively by asterisk and colon.

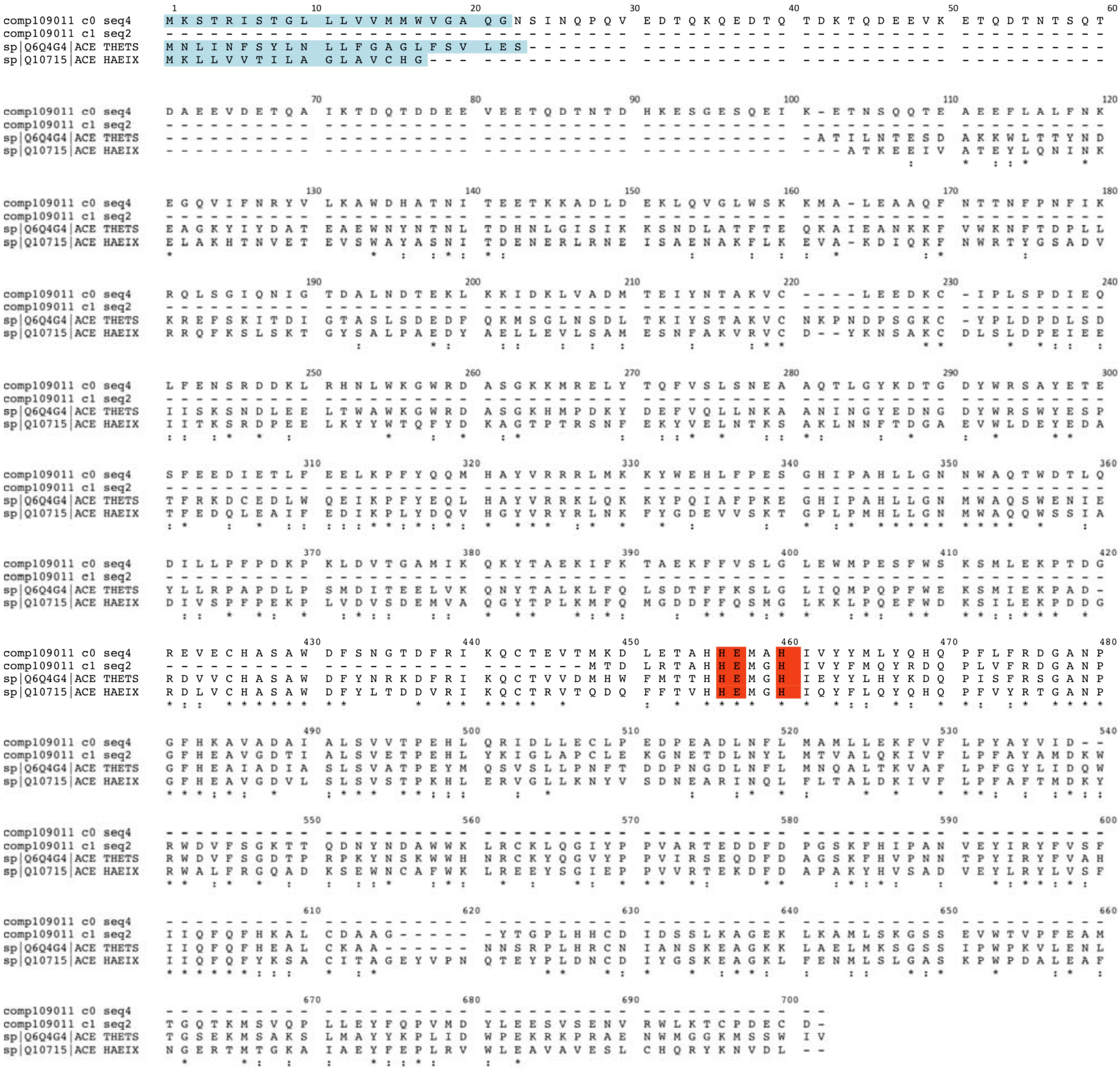

Figure S4: Alignment of the colubrarian ACE contigs with similarities to ACE with ACEs from *Theromyzon tessulatus* and *Haematobia irritans*. Signal peptides in blue and active sites in red. Identical and conserved residues are indicated respectively by asterisk and colon.

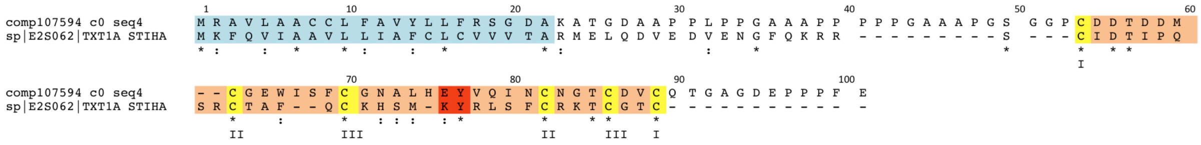

Figure S5: Alignment of the single ShKT-containing colubrarian contig with ShK Toxin from the cnidarian *Stoichodactyla heliantus.* Signal peptides in blue, ShK domain in orange, cysteines in yellow and active sites in red. Identical and conserved residues are indicated respectively by asterisk and colon. Roman numbering indicate disulfide bonds pattern.

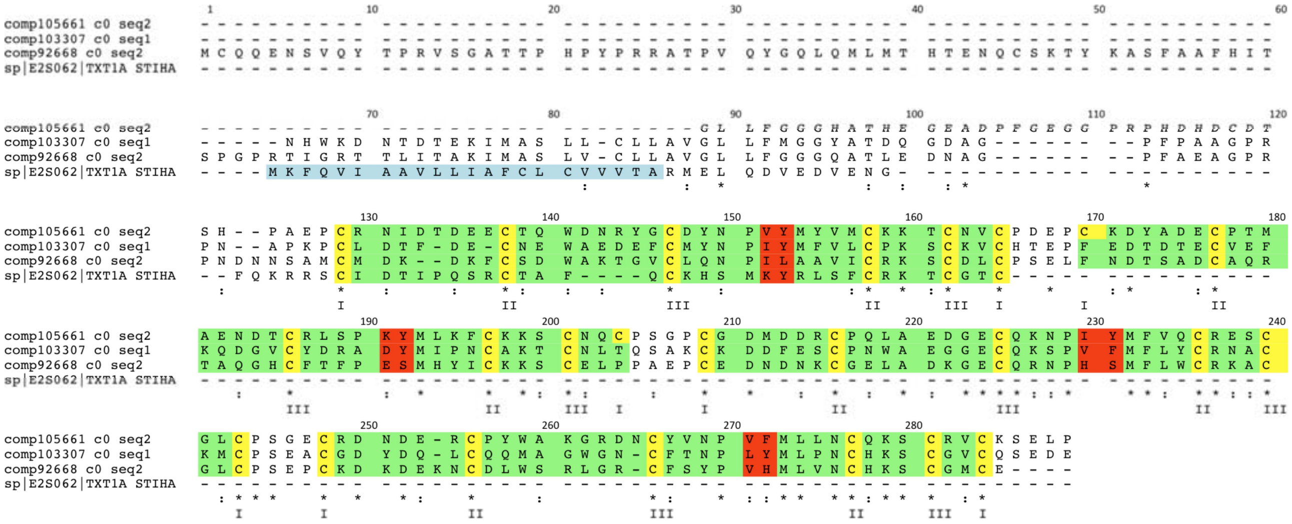

Figure S6: Alignment of the 4ShKT-containing colubrarian contig with with ShK Toxin from the cnidarian *Stoichodactyla heliantus.* Signal peptides in blue, ShK domain in green, cysteines in yellow and active sites in red. Identical and conserved residues are indicated respectively by asterisk and colon. Roman numbering indicate disulfide bonds pattern.

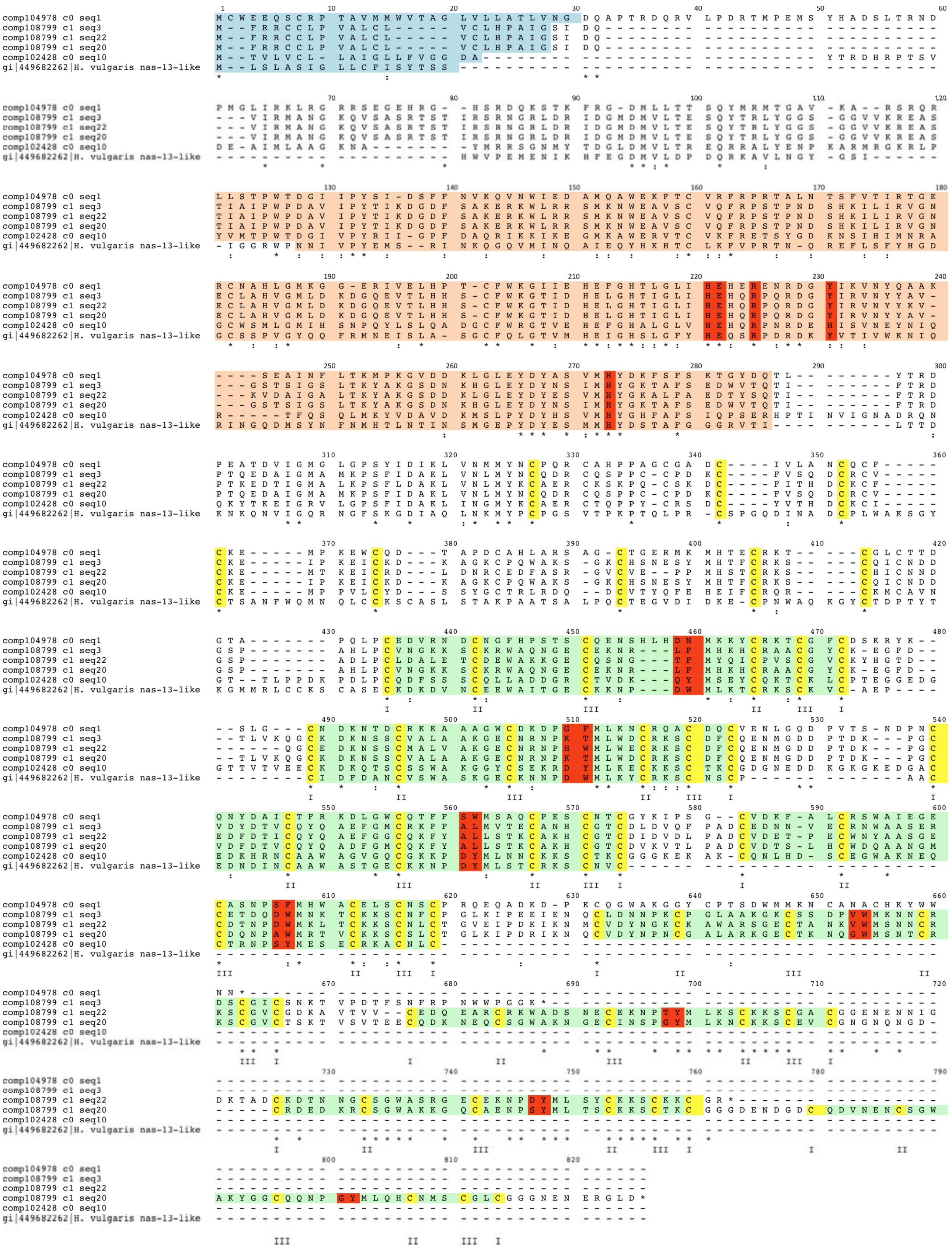

Figure S7: Alignment of the colubrarian Astacin/ShK-containing contigs with Hydra vulgaris metalloprotease nas-13-like. Signal peptides in blue, Astacin domain in orange, ShK domain in green, cysteines in yellow and active sites in red. Identical and conserved residues are indicated respectively by asterisk and colon. Roman numbering indicate disulfide bonds pattern.

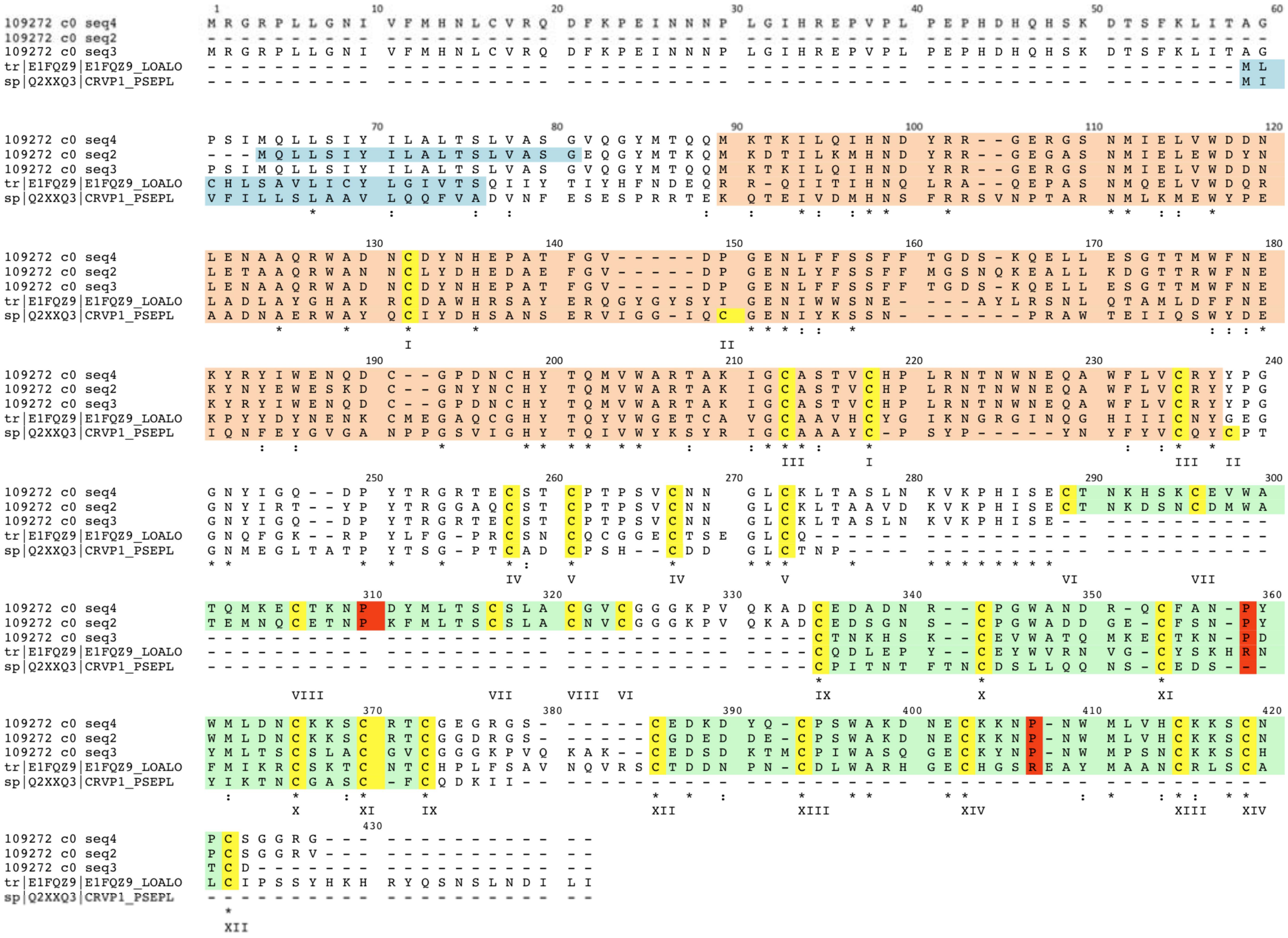

Figure S8: Alignment of the colubrarian CAP/ShK-containing contigs with the Cystein-rich venom protein ENH1 from the snake *Pseudoferania polylepis* and a similar protein form the parasitic nematode *Loa loa.* Signal peptides in blue, CAP domain in orange, ShK domain in green, cysteines in yellow and active sites in red. Identical and conserved residues are indicated respectively by asterisk and colon. Roman numbering indicate disulfide bonds pattern.

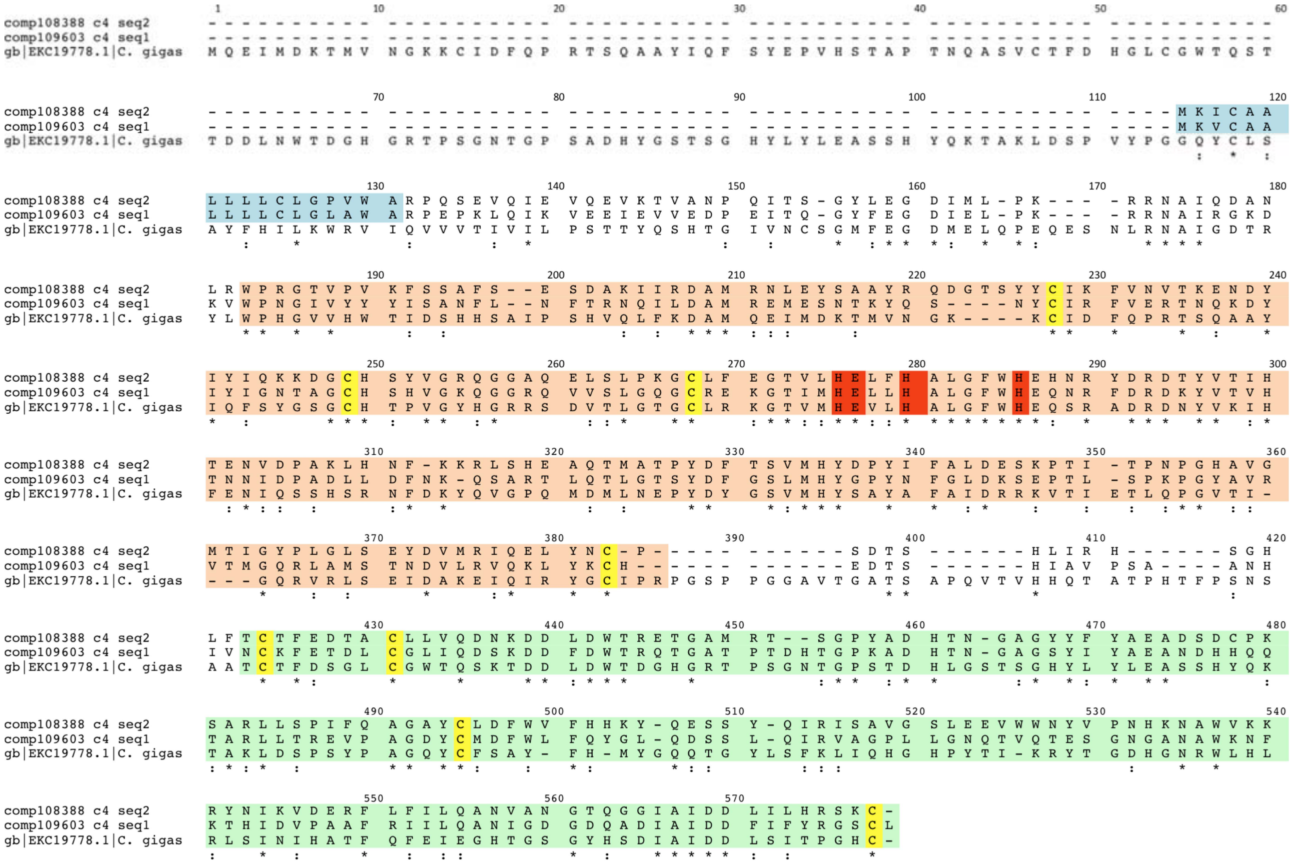

Figure S9: Alignment of the colubrarian Meprin-like contigs with Meprin A subunit B form *Crassostrea gigas*. Signal peptides in blue, Astacin domain in orange, MAM domain in green, cysteines in yellow and active sites in red. Identical and conserved residues are indicated respectively by asterisk and colon. Roman numbering indicate disulfide bonds pattern.

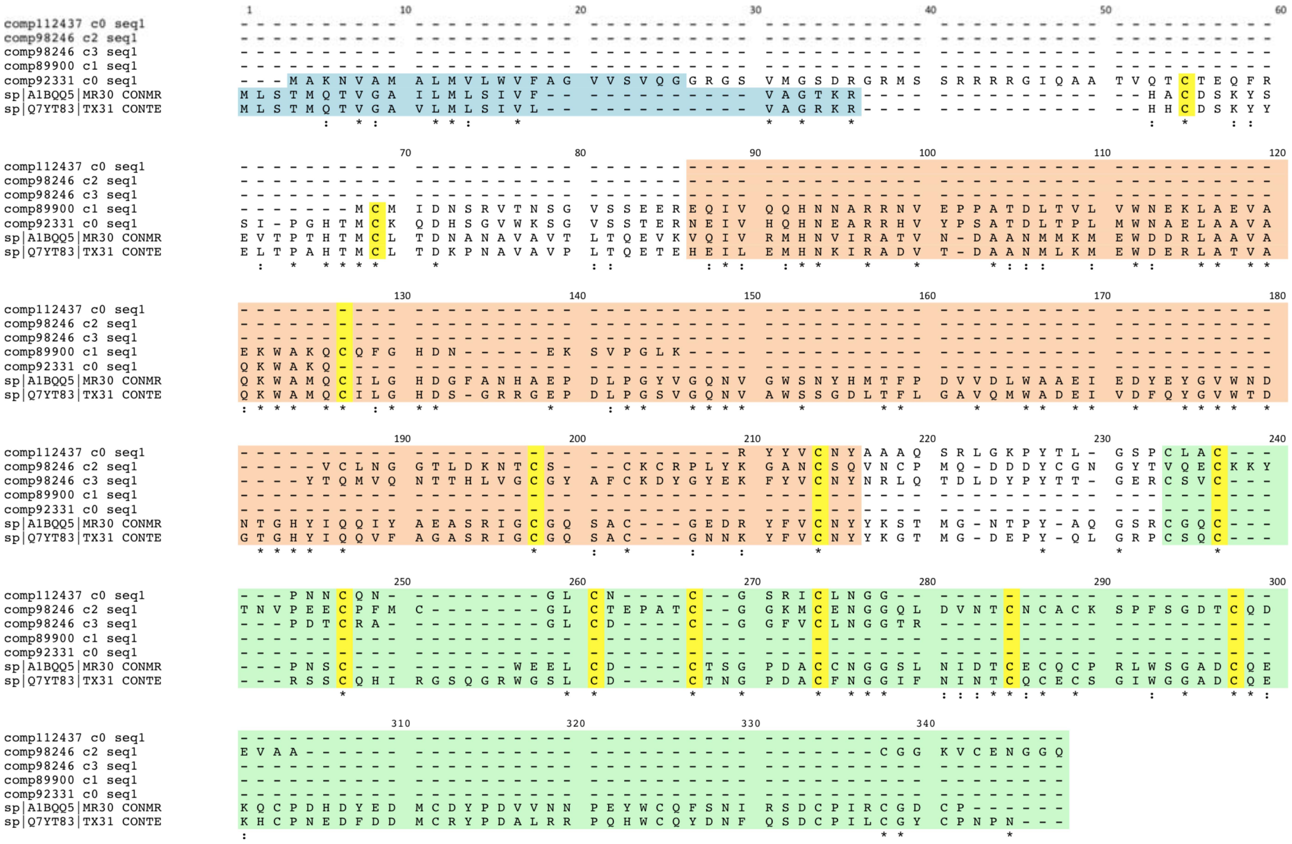

Figure S10: Alignment of the colubrarian CRISP contigs with proteins Mr30 from Conus marmoreus and Tex31 from Conus textile. Signal peptides in blue, SCP domain in orange, cysteine-rich domain in green and cysteines in yellow. Identical and conserved residues are indicated respectively by asterisk and colon.

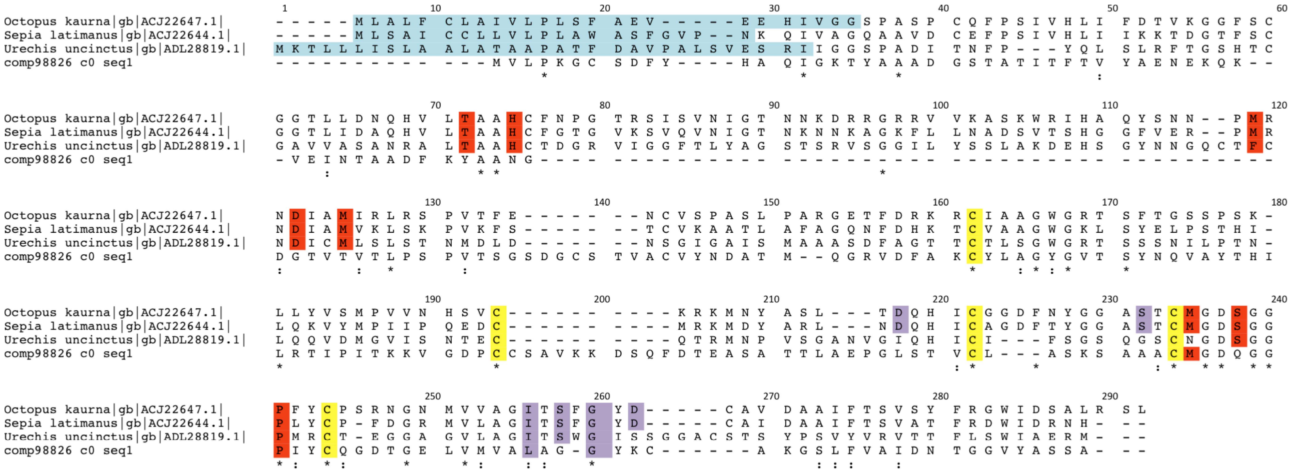

Figure S11: Alignment of the colubrarian Trypsin-like contig with trypsins from the cephalopods *Octopus kaurna* and *Sepia latimanus* and with the fibrinolytic enzyme of the echiurid *Urechis uncinctus*. Signal peptides in blue, active sites in red, substrate-binding sites in purple and cysteines in yellow. Identical and conserved residues are indicated respectively by asterisk and colon.

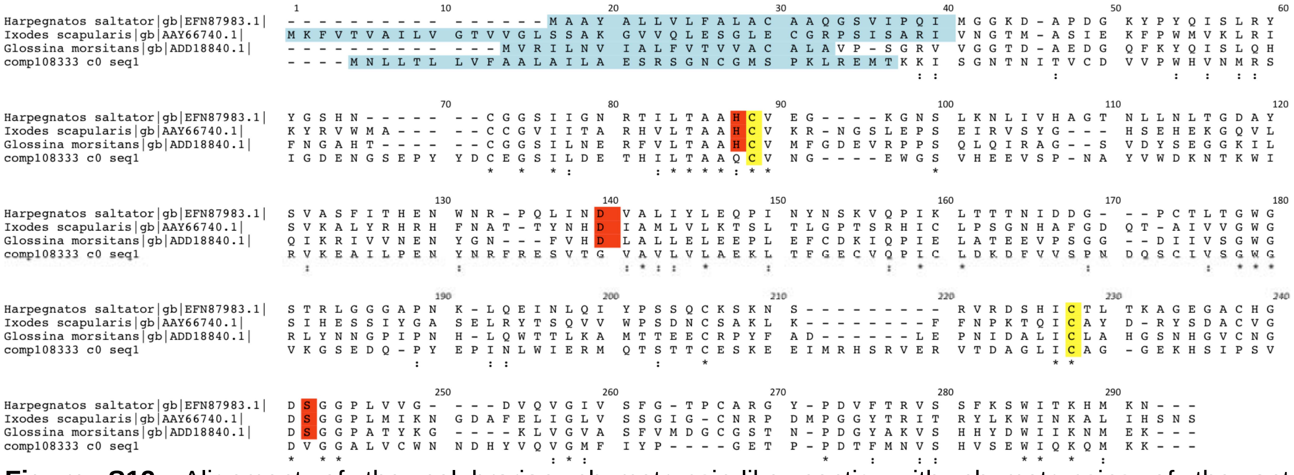

Figure S12: Alignment of the colubrarian chymotrypsin-like contig with chymotrypsins of the ant *Harpegnatos saltator* and the hematophagous arthropods *Ixodes scapularis* and *Glossina morsitans.* Signal peptides in blue, active sites in red, substrate-binding sites in purple and cysteines in yellow. Identical and conserved residues are indicated respectively by asterisk and colon.

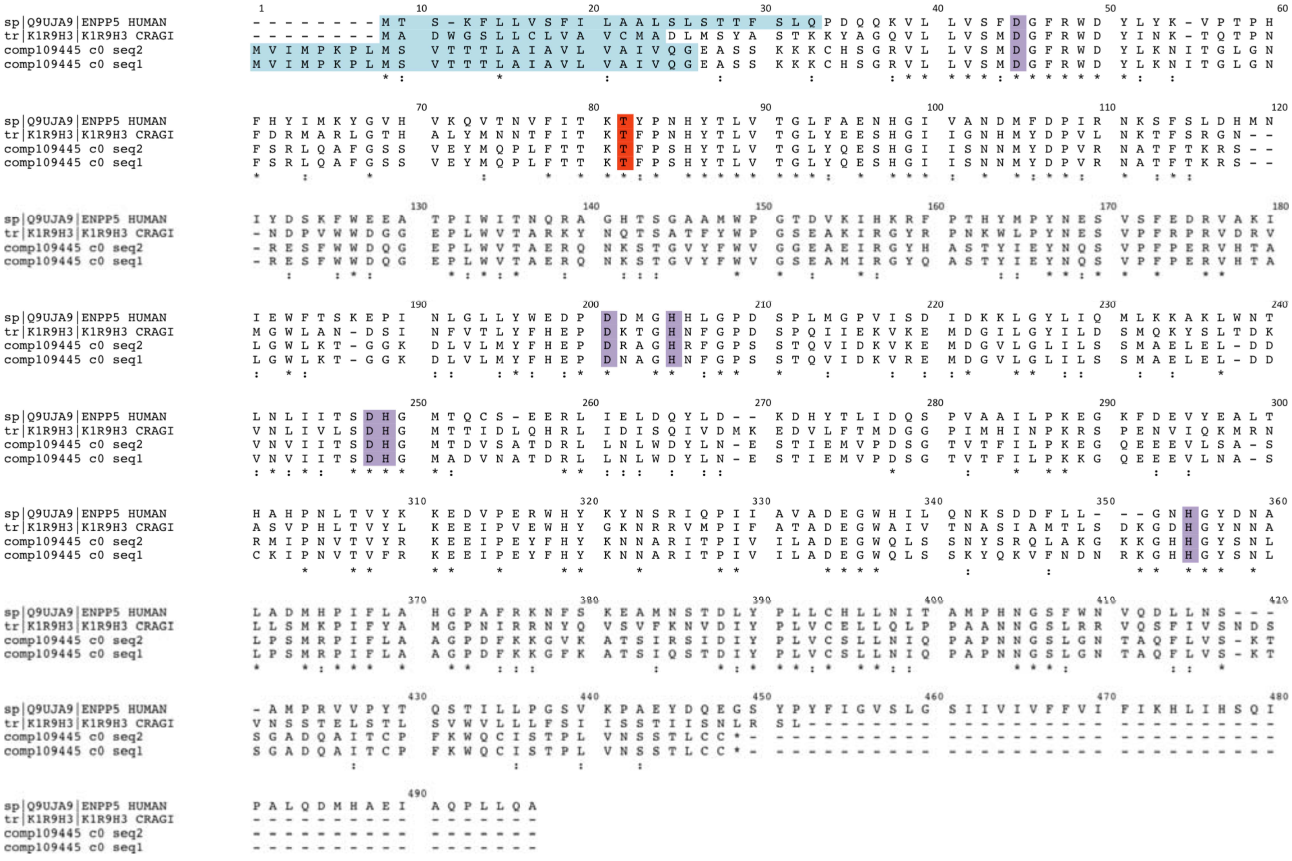

Figure S13: Alignment of the colubrarian contig of the Ectonucleotide pyrophosphatase/phosphodiesterase family 5 (ENPP5) with its human homolog and the ENPP5 from the mollusc *Crassostrea gigas.* Signal peptides in blue, active sites in red, zinc-binding sites in purple and cysteines in yellow. Identical and conserved residues are indicated respectively by asterisk and colon.

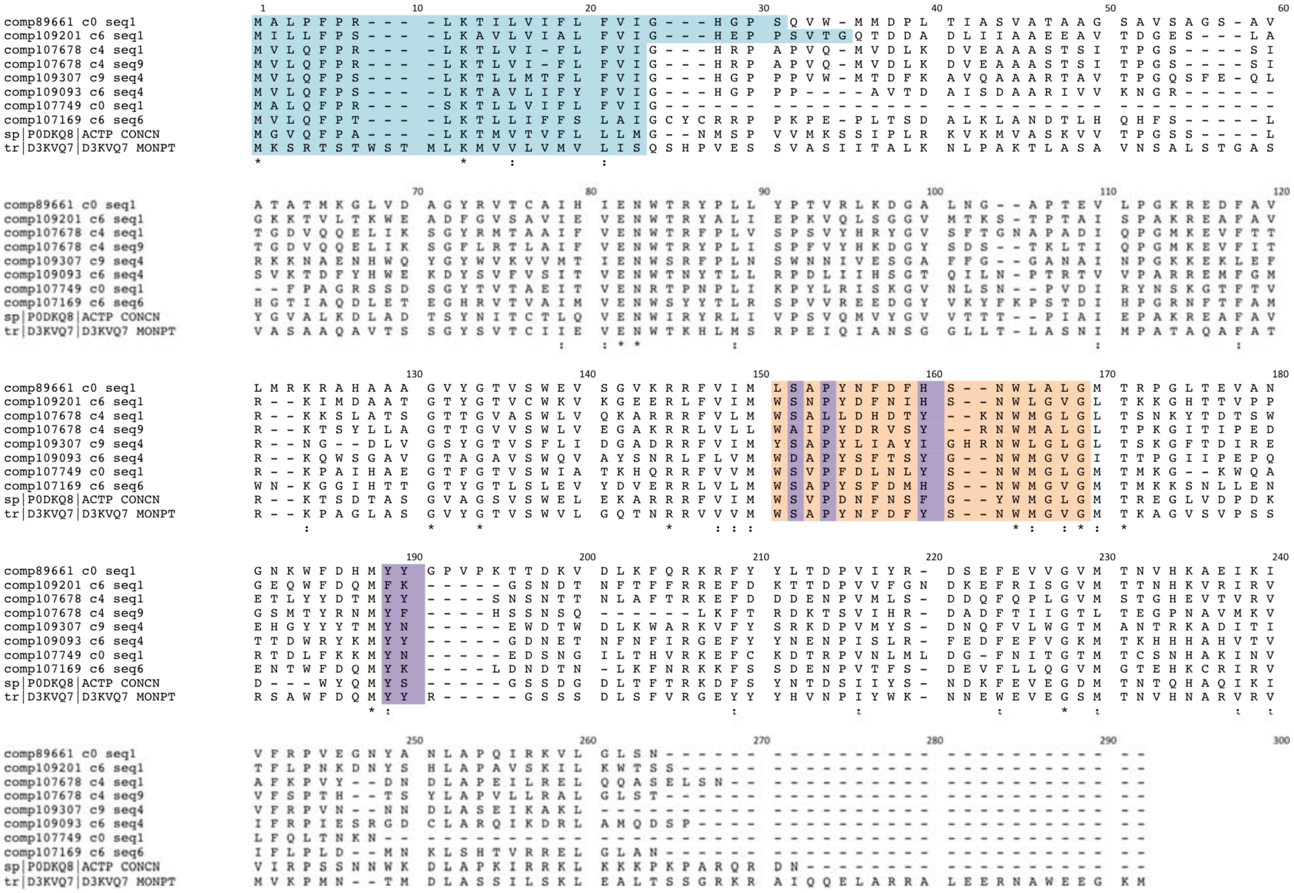

Figure S14: Alignment of the colubrarian porin contigs with echotoxin and conoporin sequences. Signal peptides in blue, Trp-rich region in orange, sites involved in the initial contact with the membrane in purple. Identical and conserved residues are indicated respectively by asterisk and colon.

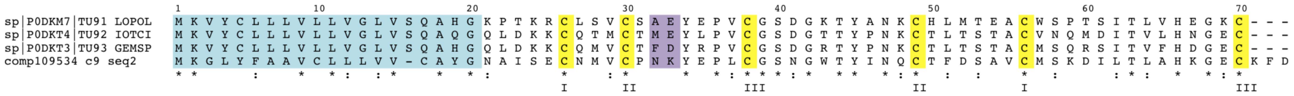

Figure S15: Figure S11: Alignment of the colubrarian turripeptide contig with its homologs from the Turridae Lophiotoma olangoensis, Iotyrris cingulifera and Gemmula speciosa. Signal peptides in blue, Kazal signature in purple and cysteines in yellow. Identical and conserved residues are indicated respectively by asterisk and colon. Roman numbering indicate disulfide bonds pattern.
